# The mechanism of *Gα_q_* regulation of *PLCβ3*-catalyzed *PIP2* hydrolysis

**DOI:** 10.1073/pnas.2315011120

**Published:** 2023-11-22

**Authors:** Maria E. Falzone, Roderick MacKinnon

**Affiliations:** ^a^Laboratory of Molecular Neurobiology and Biophysics, The Rockefeller University, New York, NY 10065; ^b^HHMI, The Rockefeller University, New York, NY 10065

**Keywords:** *PLCβ3*, *Gα_q_*, *PIP2*, GPCR signaling, *Gβγ*

## Abstract

*PLCβ* (*Phospholipase Cβ*) enzymes cleave phosphatidylinositol 4,5-bisphosphate (*PIP2)* producing *IP3* and *DAG* (diacylglycerol). *PIP2* modulates the function of many ion channels, while *IP3* and *DAG* regulate intracellular Ca^2+^ levels and protein phosphorylation by protein kinase C, respectively. *PLCβ* enzymes are under the control of G protein coupled receptor signaling through direct interactions with G proteins *Gβγ* and *Gα_q_* and have been shown to be coincidence detectors for dual stimulation of *Gα_q_* and *Gα*_*i*_-coupled receptors. *PLCβs* are aqueous-soluble cytoplasmic enzymes but partition onto the membrane surface to access their lipid substrate, complicating their functional and structural characterization. Using newly developed methods, we recently showed that *Gβγ* activates *PLCβ3* by recruiting it to the membrane. Using these same methods, here we show that *Gα_q_* increases the catalytic rate constant, *k_cat_*, of *PLCβ3*. Since stimulation of *PLCβ3* by *Gα_q_* depends on an autoinhibitory element (the X-Y linker), we propose that *Gα_q_* produces partial relief of the X-Y linker autoinhibition through an allosteric mechanism. We also determined membrane-bound structures of the *PLCβ3·Gα_q_* and *PLCβ3·Gβγ(2)·Gα_q_* complexes, which show that these G proteins can bind simultaneously and independently of each other to regulate *PLCβ3* activity. The structures rationalize a finding in the enzyme assay, that costimulation by both G proteins follows a product rule of each independent stimulus. We conclude that baseline activity of *PLCβ3* is strongly suppressed, but the effect of G proteins, especially acting together, provides a robust stimulus upon G protein stimulation.

## Significance

For certain cellular signaling processes, the background activity of signaling enzymes must be minimal and stimulus-dependent activation robust. Nowhere is this truer than in signaling by *PLCβ3* (*Phospholipase Cβ*), whose activity regulates intracellular Ca^2+^, phosphorylation by Protein Kinase C, and the activity of numerous ion channels and membrane receptors. In this study we show how *PLCβ3* enzymes are regulated by two kinds of G proteins, *Gβγ* and *Gα_q_*. Enzyme activity studies and structures on membranes show how these G proteins act by separate, independent mechanisms, leading to a product rule of costimulation when they act together. The findings explain how cells achieve robust stimulation of *PLCβ3* in the setting of low background activity, properties essential to cell health and survival.

*Phospholipase Cβ* (*PLCβ*) enzymes cleave phosphatidylinositol 4,5-bisphosphate (*PIP2)* in the plasma membrane to produce inositol triphosphate (*IP3*) and diacylglycerol (*DAG*), (1, 2). *PIP2* regulates the function of many membrane proteins including ion channels, *IP3* increases cytoplasmic Ca^2+^ via the *IP3* receptor, and *DAG* activates protein kinase C, which itself regulates numerous target proteins (3–5). Because *PIP2*, *IP3*, and *DAG* are critical to so many cellular processes, their tight regulation by *PLCβs* is essential to normal cellular function. *PLCβ* enzymes are under the control of G protein coupled receptor (GPCR) signaling through direct interaction with G proteins, *Gα_q_* and *Gβγ* (6–8). Basal activity of *PLCβs* is maintained at very low levels in cells via two autoinhibitory elements, the X-Y linker, which occupies the active site, and the Hα2’ in the proximal c-terminal domain (CTD), whose mechanism of autoinhibition is not well understood (9–13).

*PLCβs* are aqueous-soluble enzymes that must partition onto the membrane to carry out *PIP2* hydrolysis, which has posed a challenge to obtaining a quantitative description of their catalysis and regulation by G proteins. To overcome this challenge, we recently developed methods to measure both the partitioning of *PLCβ* enzymes between aqueous solution and membrane surfaces and the hydrolysis of *PIP2* by membrane-bound enzyme (14). With these methods, we showed that *PLCβ3* is a Michaelis-Menten enzyme and that *Gβγ-*dependent activation is mediated by increasing its local concentration at the membrane surface. *Gβγ* does not significantly change the catalytic rate constant (*k_cat_*) of *PLCβ3* nor does it alter its autoinhibitory elements in structures of the *PLCβ3·Gβγ(2)* complex (14).

The mechanism of activation by *Gα_q_* is not understood, particularly the potential role of the membrane in activation. Specifically, it is not clear whether *Gα_q_* activates by membrane recruitment like *Gβγ* or whether it increases *k_cat_* through an allosteric mechanism. The lipid anchor on *Gα_q_* is not required for activation of *PLCβs*, in contrast to *Gβγ*, suggesting that membrane recruitment might not underlie *Gα_q_*-dependent activation (10, 11, 15, 16). However, nonlipidated *Gα_q_* has been shown to maintain its association with membranes in cells and in vitro, raising the possibility that membrane recruitment could still play a role even in the absence of a covalent lipid group (15). It has also been established that *Gα_q_* does not activate *PLCβs* in the absence of a membrane environment, suggesting that the membrane does play a role in activation (13).

Structural studies of the *PLCβ3·Gα_q_* complex in the absence of membranes revealed that *Gα_q_* binds to the proximal and distal CTD of *PLCβ3* and *Gα_q_* binding displaces the autoinhibitory Hα2’ away from its binding site on the catalytic core by ~50 Å (10, 16). These observations led to the proposal that *Gα_q_* actives *PLCβs* by relieving Hα2’ autoinhibition. However, the mechanism of autoinhibition by Hα2’ is unknown: it is only known that removing the Hα2’ or disrupting its contacts with the catalytic core increases the basal activity of *PLCβs* (9, 11).

Some *PLCβs*, including *PLCβ3*, can also be activated by *Gβγ* and *Gα_q_* simultaneously. This dual activation, which underlies many physiological functions, was proposed to play a role in coincidence detection under costimulation of *Gα*_*i*_ and *Gα_q_*-coupled receptors in cells (8, 17, 18). Dual activation was proposed to be synergistic, or greater than the sum of the activation of each G protein on its own (19).

The goal of the present study is to understand the mechanism of activation of *PLCβ3* by *Gα_q_* and of dual activation by *Gα_q_* and *Gβγ*. Using functional experiments, membrane partitioning studies, and structural studies on membrane surfaces, we show that nonlipidated *Gα_q_* activates *PLCβ3* by increasing its catalytic rate constant, *k_cat_*, without affecting membrane recruitment. We also show that *Gα_q_*-stimulated enhancement of *k_cat_* is mediated by the X-Y linker autoinhibitory element. Thus, the X-Y linker is a suppressor of *k_cat_* that is partially relieved by *Gα_q_*. Finally, we show that nonlipidated *Gα_q_* and *Gβγ* regulate *PLCβ* function independently, the former through *k_cat_* and the latter through membrane recruitment. Consequently, dual stimulation yields activity enhancement equal to the product of the two independent stimuli.

## Results

### Activation of *PLCβ3* by Nonlipidated *Gα_q_*.

*PLCβ3* is an aqueous-soluble enzyme that partitions onto the membrane surface to catalyze *PIP2* hydrolysis. As we will describe below, we use the partition coefficient of *PLCβ3* to calculate its membrane surface concentration from its aqueous concentration set by experimental design (14). For reasons that will become apparent, we use two different concentration units in this study. In some circumstances, we specify molar concentration using the notation [*quantity*]*_molar_*. In other circumstances, we specify mole fraction (*mf*  ) in *mole%* (100 × *mf*   ) using the notation [*quantity*]. Because moles of solvent (water for aqueous solutions and lipids for membranes) are in vast excess of moles of solute (*PLCβ3* for aqueous solutions and *PLCβ3* and *PIP2* for membranes), we approximate *mf* as moles solute over moles solvent (14).

**Fig. 1.**
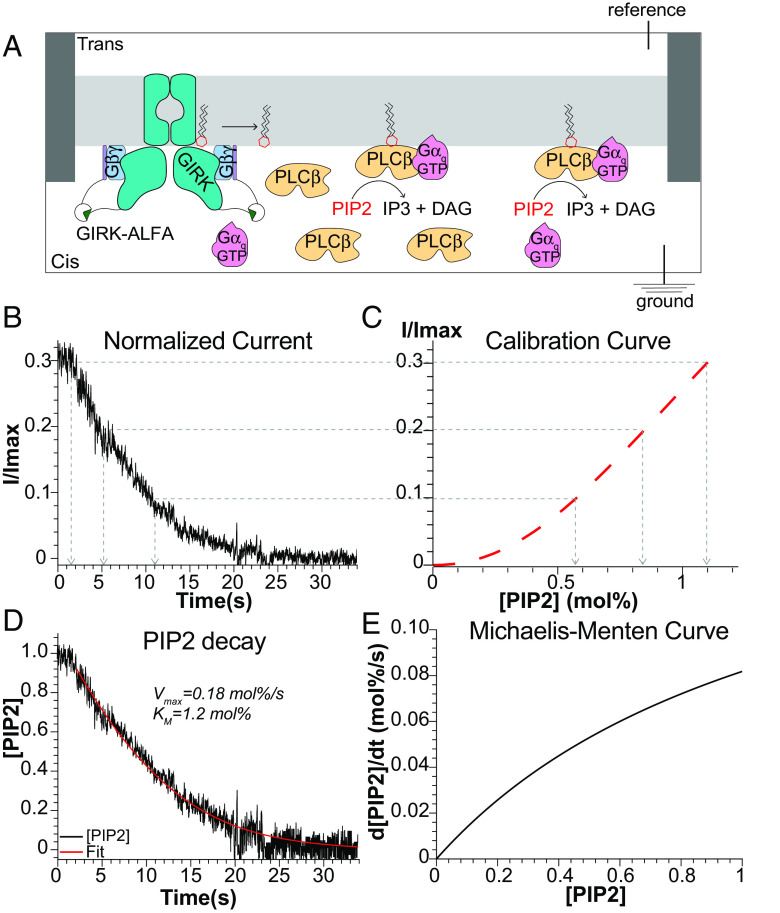
Summary of *PLCβ* functional assay and analysis. (*A*) Cartoon schematic of planar lipid bilayer setup used to measure *PLCβ3* function. (*B*–*D*) Summary of analysis of *PLCβ3*-dependent current decays. The *PIP2* calibration curve for GIRK-ALFA (*C*) is used to convert the normalized current decay (using 29 *nM* *PLCβ3* and lipidated *Gβγ*) (*B*) to *PIP2* decay (*D*) (14). Points on the normalized current decay are matched to *[PIP2]* and time. The resulting *PIP2* decay as a function of time is fit (R^2^ = 0.97) to the Lambert W Function (*SI Appendix*, Eq. S3) derived through integration of the Michaelis-Menten equation with free parameters *V_max_* and *K_M_* shown. (*E*) Corresponding Michaelis-Menten curve with the *K_M_* and *V_max_* values determined in (*D*).

To measure *PIP2* hydrolysis by *PLCβ3* on membrane surfaces, we employed an enzyme assay described in a recent publication (14). Briefly, the reaction takes place on a planar lipid bilayer formed over a hole in a partition separating two aqueous chambers (Fig. 1*A*). The lipid bilayer is composed of 2 1,2-dioleoyl-sn-glycero-3-phosphoethanolamine (DOPE):1 1-palmitoyl-2-oleoyl-glycero-3-phosphocholine (POPC):1 1-palmitoyl-2-oleoyl-sn-glycero-3-phospho-L-serine (POPS) (wt:wt:wt), plus a predetermined concentration of *PIP2* ([*PIP2*] = 1.0 *mol%*), and a *PIP2*-gated ion channel is incorporated into the bilayer via vesicle fusion. The ion channel, a modified *PIP2*-dependent, G protein-dependent inward rectifier K^+^ channel, called GIRK-ALFA, is calibrated so that the normalized K^+^ current level can be converted to membrane *PIP2* concentration (Fig. 1 *B*–*D*) (14). Upon addition of *PLCβ3* under continuous mixing, after an approximately 2 s delay, the GIRK-ALFA current decreases due to hydrolysis of *PIP2* by the added *PLCβ3* (Fig. 1*B*). Using the predetermined calibration curve (Fig. 1*C*), normalized current as a function of time (Fig. 1*B*) can be converted to *PIP2* concentration as a function of time, as shown (Fig. 1*D*). The latter curve corresponds very well (typical R^2^ > 0.9) to a Lambert W Function (aka Product Log Function) (20), which describes a linear decay initially (when *PIP2* concentration is high) and an exponential decay at later times (when *PIP2* concentration is low) (Fig. 1*D*). The Lambert W Function derives from integration of the well-known Michaelis-Menten enzyme equation,

[1] $$v=\frac{d[PIP2]}{d\tau }=-\frac{V_{\max}\left[PIP2\right]}{K_{M}+\left[PIP2\right]},$$

which, when integrated from τ = 0 to τ = t, yields

[2] $$\left[PIP2\left(t\right)\right]\;=\;K_{M}ProductLog\frac{e^{\frac{\left(\left[PIP2\left(0\right)\right]-tV_{\max}\right)}{K_{M}}}\left[PIP2\left(0\right)\right]}{K_{M}}.$$

**Fig. 2.**
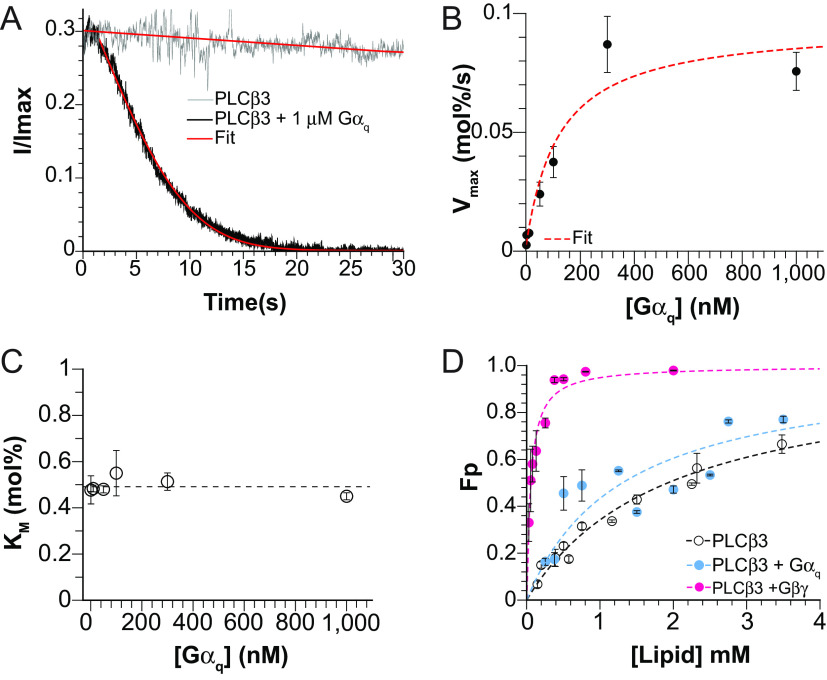
Activation of *PLCβ3* by nonlipidated *Gα_q_*. (*A*) Representative *PLCβ3*-dependent normalized current decay with 29 *nM* enzyme in the absence of *Gα_q_* (gray) or in the presence of 1.0 *μM* *Gα*_q_ (black) fit to *SI Appendix*, Eq. S3 (red curve). In the absence of *Gα*_*q*_ (gray), *V_max_* = 0.0031 *mol%/s*, *K_M_* = 0.52 *mol%* (R^2^ = 0.97). In the presence of *Gα_q_*, *V_max_* = 0.091 *mol%/s*, *K_M_* = 0.42 *mol%*, C = 0.00092, R^2^ = 0.99. (*B*) *V_max_* as a function of *Gα_q_* concentration for 29 *nM* *PLCβ3* fit to $V_{max}\left[G\alpha_{q}\right]=\left(V_{max}\left[G\alpha_{q}\to \infty \right]-V_{max}\left[G\alpha_{q}=0\right]\right)*\left(\frac{G\alpha_{q}}{G\alpha_{q}+EC_{50}}\right)+V_{max}\left[G\alpha_{q}=0\right]$  , for *V*_*max*_[*Gα_q_*→∞] and EC_50_ where *V*_*max*_[*Gα*_*q*_ = 0] is the *V_max_* in the absence of *Gα_q_*, 0.0026 *mol%/s* (14), EC_50_ = 120 *nM* and *V*_*max*_[*Gα_q_*→∞] = 0.095 *mol%/s*, R^2^ = 0.91. Individual points are from at least 3 repeats and the error bars are SEM. (*C*) *K_M_* for 29 *nM* *PLCβ3* as a function of *Gα_q_* concentration. Dashed line highlights the mean of all values. Individual points are from at least 3 repeats and the error bars are SEM. (*D*) Membrane partitioning curve for wild-type *PLCβ3* (100 *nM* and 300 *nM*) in the presence of 200 *nM* *Gα*_q_ Q209L (blue) for 2DOPE:1POPC:1POPS (wt:wt:wt) LUVs with Fraction Partitioned (*F_p_*) on the *Y* axis. Points are the average from 2 repeats for each lipid concentration and error bars are range of mean. Data were fit to Eq. **5** to determine *K_x_* (dashed blue curve). *K_x_* = 4.2 × 10^4^, R^2^ = 0.68. Data points and the fit to Eq. **5** for *PLCβ3* (100 *nM* and 300 *nM*) alone (black) and in the presence of lipidated *Gβγ* (pink) are shown for comparison, (14).

**Fig. 3.**
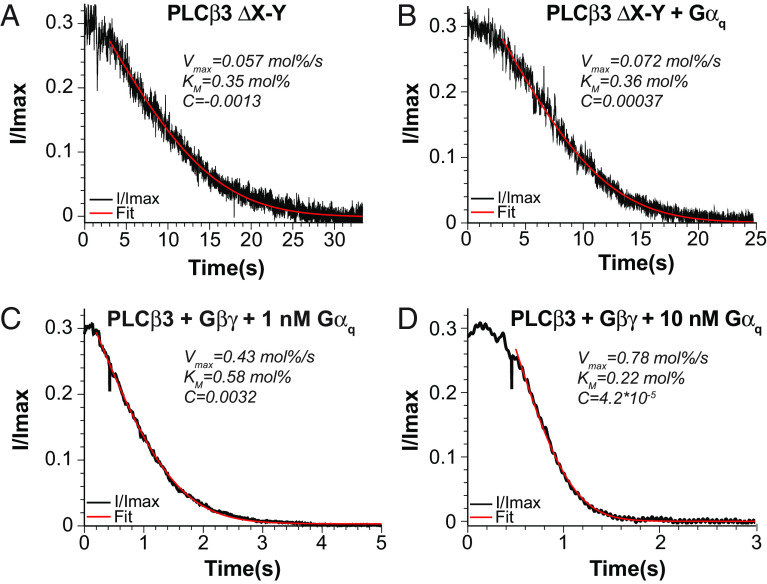
Involvement of the X-Y linker in *Gα_q_*-dependent activation and demonstration of dual activation of *PLCβ3* by *Gα_q_* and *Gβγ*. (*A* and *B*) Representative normalized current decay for *PLCβ3* lacking the entire X-Y linker (ΔX-Y all) using 290 *pM* of enzyme in the absence (*A*) or presence (*B*) of 200 *nM* *Gα_q_* fit to *SI Appendix*, Eq. S3 to determine *V_max_* and *K_M_* (red curves), R^2^ = 0.96, R^2^ = 0.98 for *A* and *B*. (*C* and *D*) Representative wildtype *PLCβ3*-dependent normalized current decay using 29 *nM* enzyme in the presence of lipidated *Gβγ* and 1.0 *nM* *Gα*_q_ (*C*) or 10 *nM* *Gα_q_* (*D*) fit to *SI Appendix*, Eq. S3 (red curve) to determine *V_max_* and *K_M_*, R^2^ = 1.0 for *C* and *D*.

An instructive description of the relationship between the Michaelis-Menten equation (Eq. **1**) and the Lambert W Function (Eq. **2**) and the suitability of the latter to our studies is given in *SI Appendix, Appendix 1*. In practice, we fit the normalized current data, i.e., Figs. 2*A* and 3 and *SI Appendix*, Fig. S2, directly with a function that is the Lambert W Function transformed by the calibration curve that converts normalized current to *PIP2* concentration (Fig. 1*C* and *SI Appendix*, Eq. S3). This function has three free parameters, *V_max_*, *K_M_*, and *C*, the latter a dimensionless (*I/I_max_*) current accounting for the small (almost inconsequential) nonspecific “leak” current observed at the longest recorded times (Figs. 1*B* and 2*A* and *SI Appendix*, Fig. S2*A*). This enzyme assay permits reproducible estimates of *V_max_* and *K_M_* for *PLCβ3* over a wide range of experimental conditions (14).

To ensure that *PLCβ3* was not affected by product inhibition under our assay conditions, we tested its function in the presence of *Gβγ* and 1.0 *mol%* *DAG* or 1.0 *μM* *IP3* (*SI Appendix*, Fig. S3). Current decays and determined values for *V_max_* and *K_M_* are indistinguishable from experiments without *DAG* and *IP3* (*SI Appendix*, Fig. S3), indicating that *PLCβ3* is not inhibited by the products of its catalyzed reaction in our experimental setup.

To study the activation of *PLCβ3* by *Gα_q_* we used nonlipidated *Gα_q_* (10, 11, 15). Because the GTP bound form of *Gα_q_* is required to activate *PLCβ3*, we used a hydrolysis-deficient mutant (Q209L) that remains constitutively bound to GTP (*SI Appendix*, Fig. S1*C*). Activation by this mutant is similar to wild-type *Gα_q_* (21–24), and migration on a size exclusion column as a complex with *PLCβ3* is indistinguishable from wild-type *Gα_q_* (*SI Appendix*, Fig. S1 *A* and *B*). When *Gα_q_* is added to the enzyme assay in the absence of *PLCβ3*, it does not affect GIRK-ALFA current (*SI Appendix*, Fig. S1*D*).

Fig. 2*A* shows the influence of nonlipidated *Gα_q_* on *PIP2* hydrolysis. In the presence of 29 *nM* *PLCβ3* in aqueous solution, the decay of GIRK-ALFA current is slow (reduction of ~15% over 30 s), reflecting slow hydrolysis of *PIP2*. In the presence of 1.0 *μM* *Gα_q_*, by contrast, the current reduction is faster, reflecting more rapid hydrolysis. The red curves correspond to fits to *SI Appendix*, Eq. S3 and yield *V_max_* = 0.0031 *mol%/s*, *K_M_* = 0.52 *mol%* (R^2^ = 0.97) in the absence of *Gα_q_* and *V_max_* = 0.091 *mol%/s*, *K_M_* = 0.42 *mol%* (R^2^ = 0.99) in the presence of 1.0 *μM* *Gα*_q_. By performing these experiments in multiples, with different concentrations of *Gα_q_* (0 to 1,000 *nM*) in the aqueous solution that interfaces the lipid bilayer, we observe that *Gα_q_* increases *V_max_* without affecting *K_M_* (Fig. 2 *B* and *C* and *SI Appendix*, Fig. S2 *A*–*F*). The red dashed curve in Fig. 2*B* is a rectangular hyperbola with a half activation concentration for *Gα_q_* about 120 *nM*. The maximum increase in *V_max_* elicited by *Gα_q_* is about 35-fold above *V_max_* in the absence of *Gα_q_*. Previous work showed a 20 to 50-fold enhancement of PLC*β3* catalysis, but any relationship to *V_max_* or *K_M_* was unknown in earlier studies of *Gα_q_* (9–11). We note that the effect of *Gα_q_* to increase *V_max_* without affecting *K_M_* is exactly what we observed for *Gβγ* stimulation of *PLCβ3* (14). However, as we will show below, the origins of these apparently similar effects are mechanistically distinct.

### *Gα_q_* Modifies *k_cat_* of *PLCβ3* Catalysis.

Because *PLCβ3* is an aqueous-soluble enzyme that must partition onto the membrane surface to catalyze *PIP2* hydrolysis, the kinetic parameter *V_max_* is the product of two separate quantities, expressible as

[3] $$V_{\max}={[PLC\beta 3}_{m}]*k_{\mathrm{cat}},$$

where [*PLCβ3_m_*] is the local mole fraction concentration of *PLCβ3* on the membrane surface (subscript m) and *k_cat_* is the first-order rate constant for the hydrolysis of *PIP2* by a *PLCβ3·PIP2* complex on the membrane surface. In principle, to increase *V_max_*, *Gα_q_* could affect either or both quantities. It is not clear whether the nonlipidated *Gα_q_* used in our experiments retains membrane binding (10, 11, 15, 16). To examine whether nonlipidated *Gα_q_* affects the membrane concentration of *PLCβ3*, we measured whether *Gα_q_* changes the degree to which *PLCβ3* partitions onto the membrane, i.e., whether *Gα_q_* recruits *PLCβ3* to the membrane surface. The concentration of *PLCβ3* at the membrane is determined by its partition coefficient, *K_x_*, which is the ratio of the mole fraction *PLCβ3* on the membrane [*PLCβ_m_*] to the mole fraction of *PLCβ3* in aqueous solution [*PLCβ_w_*]:

[4] $$K_{x}=\frac{\left[{PLC\beta }_{m}\right]}{\left[{PLC\beta }_{w}\right]}.$$

We used a vesicle spin-down assay to measure the fraction of *Gα_q_* or *PLCβ3* in the absence and presence of *Gα_q_* that binds to the membrane (*F_p_*). This was done by incubating large unilamellar vesicles (LUVs) consisting of 2DOPE:1POPC:1POPS (wt:wt:wt) with *Gα_q_* or *PLCβ3* (±*Gα_q_*), centrifuging the mixture, and then measuring the fraction of *Gα_q_* or *PLCβ3* associated with the membranes (*SI Appendix*, Fig. S4 *A*–*C*). These experiments were carried out at several lipid concentrations and the measured *F_p_* for *Gα_q_* or *PLCβ3* (±*Gα_q_*) was graphed as a function of lipid concentration (Fig. 2*D* and *SI Appendix*, Fig. S4 *E*–*G*). The dashed curves correspond to the function

[5] $$\begin{array}{c} F_{p}=\frac{\left[{PLC\beta 3}_{m}\right]{\left[L\right]}_{\mathrm{molar}}}{\left[{PLC\beta 3}_{m}\right]{\left[L\right]}_{\mathrm{molar}}+\left[{PLC\beta 3}_{w}\right]{\left[W\right]}_{\mathrm{molar}}}\\ \;=\frac{K_{x}{\left[L\right]}_{\mathrm{molar}}}{K_{x}{\left[L\right]}_{\mathrm{molar}}+{\left[W\right]}_{\mathrm{molar}}}, \end{array}$$

where [*W*]*_molar_* is the molar concentration of water, ~55 *M*, and [L]_molar_ is half the total lipid concentration, recognizing that proteins can only access the outer leaflet of the LUVs. Therefore, *K_x_* is the only free parameter (Fig. 2*D* and *SI Appendix*, Fig. S4 *F* and *G*) (14, 25). The results show that neither wildtype nor the Q209L mutant *Gα_q_* affect partitioning of *PLCβ3* onto these membrane surfaces (Fig. 2*D* and *SI Appendix*, Fig. S4 *B*, *C*, and *F*). Moreover, *Gα_q_* alone, at least the nonlipidated version used in these experiments, does not partition onto membranes in our experiments, in contrast to previously reported results (15) (*SI Appendix*, Fig. S4 *A* and *E*).

Having established that nonlipidated *Gα_q_* used in these studies does not increase the membrane-bound concentration of *PLCβ3*, from Eq. **3** we conclude that the increase in *V_max_* in the presence of *Gα_q_* must come from an increased *k_cat_*. In the experiments shown in Fig. 2*B*, the aqueous concentration of *PLCβ3* was 29 *nM* = 5.3 × 10^−8^ *mol%*, which, using the partition coefficient *K_x_* = 2.9 × 10^4^ (14) and Eq. **4**, yields a membrane concentration for *PLCβ3*, [*PLCβ_m_*] = 1.5 × 10^−3^ *mol%*. Therefore, from *V_max_* = 0.091 *mol%/s* (Fig. 2*A*) and Eq. **3**, we calculate *k_cat_* ~60 *s*^*−1*^ in the presence of a maximally activating concentration of *Gα_q_*, which is about 35-fold higher than *k_cat_*in the absence of *Gα_q_* (Fig. 2*A* and Table 1) (14). This finding contrasts the influence of *Gβγ* on *PLCβ3* function, which increases *V_max_* almost entirely through membrane recruitment with little effect on *k_cat_* (Table 1) (14). We note that in the cellular environment where *Gα_q_* is lipidated and membrane-associated, it is likely to also increase [*PLCβ_m_*] in addition to the established increase in *k_cat_*.

**Table 1.** Kinetic parameters for *PLCβ3*

Condition	*K*_M_ (*mol%*)	*k*_cat_ (*s^−1^*)	Fold-increase^*^
*PLCβ3* alone^†^	0.43 ± 0.05	1.7 ± 0.5	-
*PLCβ3* + lipidated *Gβγ*^†^	0.42 ± 0.05	3.2 ± 0.5	1.9
*PLCβ3* + *Gα_q_*^‡^	0.51 ± 0.04	56.9 ± 8	34
*PLCβ3* ΔX-Y all	0.33 ± 0.04	1,977.5 ± 150	1,160
*PLCβ3* ΔX-Y all + *Gα_q_*^§^	0.31 ± 0.02	2,485 ± 250	1,460
*PLCβ3* ΔX-Y contact	0.36 ± 0.02	588.5 ± 90	346
*PLCβ3* ΔX-Y contact + *Gα_q_*^§^	0.34 ± 0.04	1,161.3 ± 140	679

^*^Over wild-type basal activity.

^†^Previously determined (14).

^‡^For saturating *Gα_q_*, 300 *nM*.

^§^For 200 *nM* *Gα_q_*.

Our observation that *Gα_q_* increases *k_cat_* without discernably affecting *K_M_* places constraints on the rate constants of a Michaelis-Menten kinetic reaction scheme. Specifically, for the reaction (PLCβ3 + $PIP2\overset{k_{1}}{\underset{k_{-1}}{⇆}}PLC\beta 3\cdot PIP2\overset{k_{cat}}{\to }PLC\beta +\;IP3+DAG)$ , where *k_1_* and *k_−1_* are the forward and reverse rate constants for *PLCβ3·PIP2* complex formation and *k_cat_* the catalytic step, *K_M_* is

[6] $$K_{M}=\frac{k_{-1}+k_{\mathrm{cat}}}{k_{1}}.$$

The most likely explanation for a 35-fold change in *k_cat_* with little effect on *K_M_* is that *k_−1_* >> *k_cat_* so that the value of the numerator is little affected by changes in the smaller quantity, *k_cat_*. In the framework of the above reaction scheme, this would imply that most encounters between *PLCβ3* and *PIP2* dissociate prior to hydrolysis.

### *Gα_q_*-Dependent Activation Is Dependent on the X-Y Linker.

A natural explanation for how *Gα_q_* increases *k_cat_* is that it somehow destabilizes the interaction between the autoinhibitory X-Y linker and the active site, allowing it to be displaced with a higher probability. To test this possibility, we expressed and purified *PLCβ3* lacking the entire X-Y linker (R471-V584, *PLCβ3* ΔX-Y all) or the segment of the linker in direct contact with the active site (T575-V584, ΔX-Y contact) and tested their basal and *Gα_q_*-dependent catalytic activity. If *Gα_q_*-dependent activation is mediated through the X-Y linker, then the maximum fold-activation by *Gα_q_* should be significantly reduced, which has been previously reported (12, 13, 26).

Both X-Y linker mutants exhibited significantly increased basal (i.e., unstimulated by G proteins) *V_max_*: ~2,300-fold for *PLCβ3* ΔX-Y all and ~700-fold for *PLCβ3* ΔX-Y contact, consistent with the autoinhibitory function of the linker (Fig. 3*A* and *SI Appendix*, Fig. S2*G*). Membrane partitioning experiments showed that membrane association is enhanced only ~two-fold in the ΔX-Y all construct (*SI Appendix*, Fig. S4 *D* and *G*). Therefore, the increase in basal activity is primarily due to an increase in *k_cat_*, ~1,100-fold for *PLCβ3* ΔX-Y all and ~350-fold for *PLCβ3* ΔX-Y contact (Table 1). This observation also indicates that partitioning is not significantly influenced by the X-Y linker. In addition, the *K_M_* values for the deletion mutants were not significantly different than wildtype (Table 1), suggesting that the linker does not simply act as a competitive inhibitor, blocking *PIP2* from binding to the active site. The small difference in basal activity between the two constructs, ~three-fold, suggests that most of the autoinhibitory impact is mediated by the residues in direct contact with the active site.

Addition of 200 *nM* *Gα_q_*, which produces a ~20-fold increased *V_max_* in wild-type *PLCβ3* (Fig. 2*B*), had less than a two-fold effect on *V_max_* for *PLCβ3* ΔX-Y and ~two-fold for *PLCβ3* ΔX-Y contact (Fig. 3*B*, *SI Appendix*, Fig. S2*H*, and Table 1). Thus, an intact autoinhibitory X-Y linker is required for *Gα_q_*-dependent activation. Because the lack of *Gα_q_*-dependent activation is comparable in the two mutants, stimulation by *Gα_q_* is likely mediated through the residues that directly contact the active site. Taken together, these results suggest that the presence of the X-Y linker in the active site is a major suppressor of *k_cat_* and that *Gα_q_*-dependent activation is mediated through partial relief of this suppression.

One might wonder whether the relative insensitivity of the catalytic rate to *Gα_q_* in the ΔX-Y mutants could reflect *PIP2* depletion near the active site owing to the relatively high catalytic rates in these mutants, i.e., substrate access becomes diffusion-limited. Based on a calculation presented in *SI Appendix, Appendix 2*, we think this is unlikely to be the case. More likely, allosteric regulation of the active site of *PLCβ3* is mediated at least in part through the inhibitory X-Y linker and manifests kinetically through the altered *k_cat_* that we observe.

### Simultaneous Activation of *PLCβ3* by *Gα_q_* and *Gβγ*.

We have demonstrated that nonlipidated *Gα_q_* and lipidated *Gβγ* activate *PLCβ3* through different mechanisms, *Gβγ* through membrane recruitment to increase the membrane concentration of enzyme and *Gα_q_* by increasing the catalytic rate constant. Given these observations, we suspected that dual activation of *PLCβ3* by both G proteins would combine both mechanisms, which would lead to a product, rather than a sum, of the two effects (Eq. **3**). To test this idea, we measured *PLCβ3* activity in the presence of a high concentration of lipidated *Gβγ* and 1.0 *nM* or 10 *nM* *Gα*_q_ (Fig. 3 *C* and *D*). The current decays in the bilayer enzyme assay were very rapid, but still well fit by the transformed Lambert W Function, thus permitting determination of *V_max_* and *K_M_* (Fig. 3 *C* and *D*). Fig. 3 *C* and *D* show that *Gα_q_* induces a concentration-dependent increase in *V_max_* in the presence of *Gβγ*, as was observed in the absence of *Gβγ* (Table 2). Moreover, the fold-increase of *V_max_* in the presence of *Gα_q_* compared to that in the absence of *Gα_q_* is approximately the same whether *Gβγ* is present or not (Table 2). This supports the independent action of *Gα_q_* and *Gβγ* and the conclusion that together both G proteins increase *V_max_* by a produce rule.

**Table 2.** Effect of dual activation with *Gα_q_* and *Gβγ* on *V_max_*

Condition	*V_max_* (*mol%/s*)	Total fold-increase	Fold-increase over 0 *Gα_q_*
*PLCβ3* alone^*^	0.0026 ± 0.0007	-	-
*PLCβ3* + 1.0 *nM* *Gα_q_*	0.0068 ± 0.0007	2.6 ± 0.3	2.6 ± 0.3
*PLCβ3* + 10 *nM* *Gα_q_*	0.0076 ± 0.001	2.9 ± 0.4	2.9 ± 0.4
*PLCβ3* + Gβγ^*^	0.17 ± 0.02	65	-
*PLCβ3* + *Gβγ* + 1.0 *nM* *Gα_q_*	0.34 ± 0.1	129 ± 38	1.8 ± 0.5
*PLCβ3* + *Gβγ* + 10 *nM* *Gα_q_*	0.55 ± 0.1	213 ± 44	2.9 ± 0.6

^*^Previously reported (14).

### Structure of the *PLCβ3·Gα_q_* Complex on Lipid Vesicles.

**Fig. 4.**
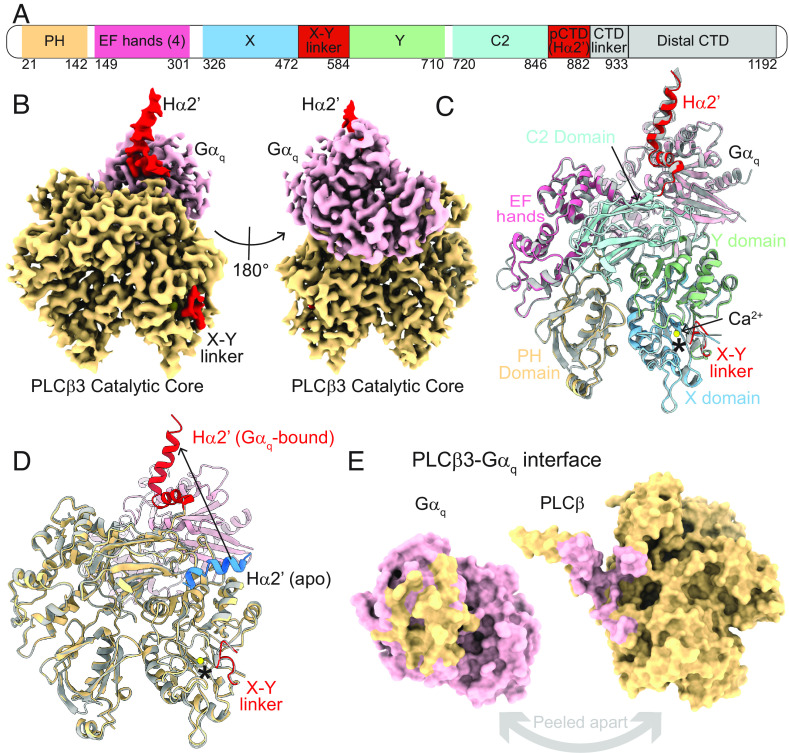
Structure of the *PLCβ3·Gα_q_* complex on lipid vesicles. (*A*) Primary structure arrangement of *PLCβ3* enzymes. Sections are colored by domain. The PH domain is yellow, the EF hand repeats are pink, the C2 domain is light teal, the Y domain is green, the X domain is light blue, the X-Y linker and the pCTD are red. Domains in gray (CTD linker and Distal CTD) are not observed in our structures. pCTD is proximal CTD. (*B*) Sharpened, masked map of the *PLCβ3·Gα_q_* complex colored by protein. *PLCβ3* is yellow and *Gα_q_* is pink. The autoinhibitory elements in *PLCβ3*, the X-Y linker and the pCTD, are colored red. (*C*) Structural alignment of the *PLCβ3·Gα_q_* complex on membranes, colored by domain as in *A*, with the previously determined crystal structure of the complex [PDBID: 4GNK, (10)], in gray. Cα rmsd is 0.84 Å. Calcium ion from the cryo-EM structure is shown as a yellow sphere and the active site is denoted with an asterisk. (*D*) Structural alignment of *PLCβ3·Gα_q_* complex on membranes, colored by protein-*PLCβ3* is yellow and *Gα_q_* is pink, with the previously determined cryo-EM structure of the apo catalytic core [PDBID: 8EMV, (14)] colored in gray. The X-Y linker and the pCTD from the *Gα_q_* complex are colored red, the calcium ion from the cryo-EM structure is shown as a yellow sphere and the active site is denoted with an asterisk. The Hα2’ from the apo structure is colored in blue to highlight its position on the catalytic core and an arrow denotes the *Gα_q_*-dependent movement of the Hα2’. (*E*) Surface representation of the *PLCβ3·Gα_q_* interface peeled apart to show extensive interactions. Residues on *PLCβ3* that interact with *Gα_q_* are colored in pink and residues on *Gα_q_* that interact with *PLCβ3* are colored in yellow. Interface residues were determined using the ChimeraX interface feature using a buried surface area cutoff of 15 Å^2^.

We determined the structure of the *PLCβ3·Gα_q_* complex associated with lipid vesicles at 3.4 Å (Fig. 4 and *SI Appendix*, Fig. S5 and Table S1). The sample was prepared by combining *PLCβ3* and wildtype *Gα_q_* bound to GDP-AlF_4_, purifying the complex using size exclusion chromatography (*SI Appendix*, Fig. S1*A*), and then mixing the purified complex with lipid vesicles composed of 2DOPE:1POPC:1POPS (wt:wt:wt). The structure of the complex contains density for the *PLCβ3* catalytic core and the proximal CTD, but the CTD linker and the distal CTD are disordered (Fig. 4 *A* and *B*), suggesting conformational heterogeneity of the domains with respect to each other. The overall complex is very similar to the previously determined crystal structure, including the X-Y linker engaged in the active site, with a Cα rmsd of 0.84 Å (10) (Fig. 4 *B* and *C*). Despite the disordered distal CTD, which was previously shown to be part of the *PLCβ3·Gα_q_* interface (10), the interface between the *PLCβ3* catalytic core and *Gα_q_* is extensive, burying ~1,500 Å^2^ and involving 56 residues, 27 from *Gα_q_* and 29 from *PLCβ3* (Fig. 4*E* and *SI Appendix*, Fig. S7*A* and Table S2 and S3). Compared to the structure of the catalytic core in the absence of *Gα_q_*, the only conformational difference is the displacement of the Hα2’ away from the catalytic core (Fig. 4*D*). Despite its proximity to the catalytic site, the Hα2’ displacement does not induce additional changes in that region (Fig. 4*D*). Membrane association of the complex also does not produce conformational differences other than the additional heterogeneity between the catalytic core and the distal CTD (Fig. 4*C*).

Despite our finding that the X-Y linker is involved functionally in *Gα_q_*-dependent activation, we do not observe structural differences at the active site or its interface with the X-Y linker, even with extensive classification targeting that region. This observation is not too surprising, however, given the magnitude of activation of *Gα_q_* compared to the activity in the absence of linker. The basal *k_cat_* and maximal *Gα_q_*-stimulated *k_cat_* are only ~0.09% and ~3%, respectively, of the activity in the absence of the linker, suggesting that *Gα_q_* does not alter the probability of its occupancy in the active site enough to be observable in structural experiments. In other words, if we take the activity in the absence of the linker as zero occupancy of the linker in the active site, then even in the presence of saturating *Gα_q_*, the linker would only be displaced 3% of the time, which is not easily detectable using cryo-EM.

### Structure of the *PLCβ3·Gβγ(2)·Gα*_q_ Complex on Lipid Vesicles.

**Fig. 5.**
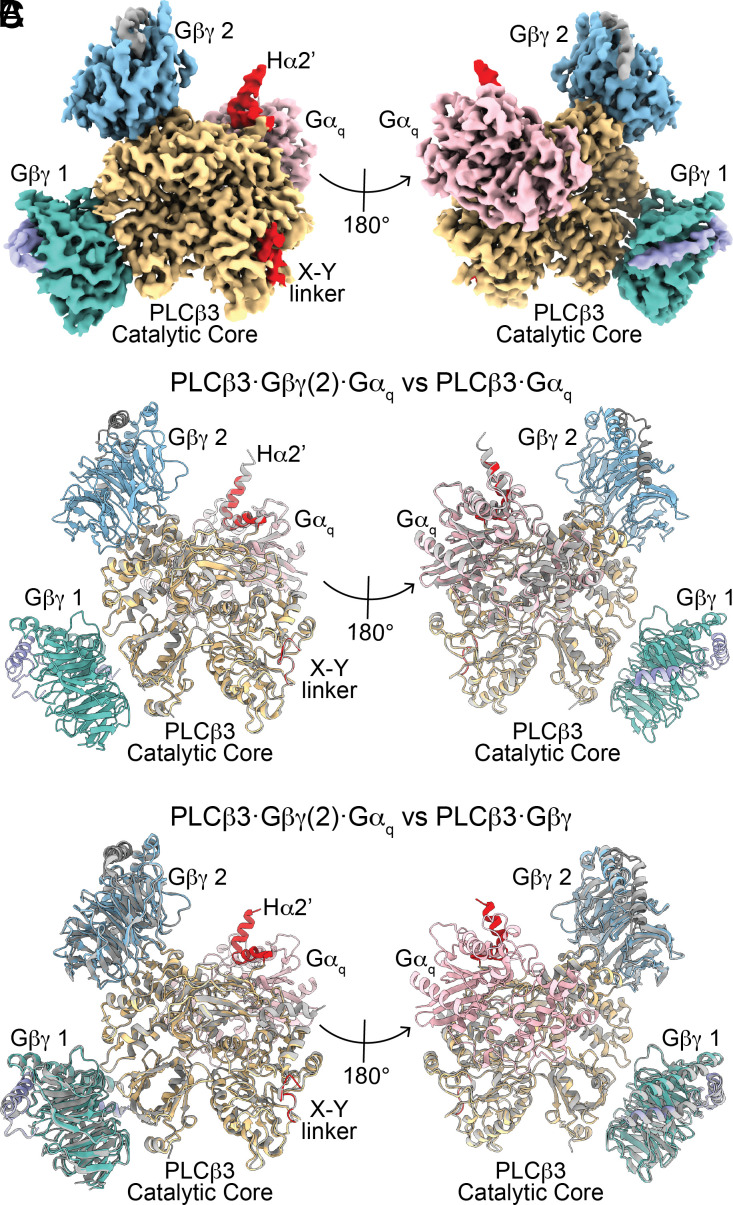
Structure of the *PLCβ3·Gβγ(2)·Gα_q_* complex on lipid vesicles. (*A*) Sharpened, masked map of the *PLCβ3·Gβγ(2)·Gα_q_* complex colored by protein. *PLCβ3* is yellow, *Gα_q_* is pink, *Gβ* 1 is dark teal, *Gγ* 1 is purple, *Gβ* 2 is light blue, *Gγ* 2 is gray. The X-Y linker and pCTD are colored red. (*B* and *C*) Structural alignment of *PLCβ3·Gβγ(2)·Gα_q_* complex on lipid vesicles, colored as in *A*, with the *PLCβ3·Gα_q_* complex on lipid vesicles in gray (*B*, Cα rmsd = 0.62 Å) or with the *PLCβ3·Gβγ(2)* complex on lipid vesicles in gray (*C*, Cα rmsd = 0.63 Å), [PDBID: 8EMW, (14)].

We also determined the structure of the *PLCβ3·Gβγ(2)·Gα_q_* complex bound to lipid vesicles to 3.4 Å resolution (Fig. 5 and *SI Appendix*, Fig. S6 and Table S1). We reconstituted lipidated *Gβγ* into vesicles comprised of 2DOPE:1POPC:1POPS (wt:wt:wt) as previously described (14), mixed *PLCβ3*, and wildtype *Gα_q_* bound to GDP-AlF_4_ and added the complex to the *Gβγ*-containing lipid vesicles for grid preparation. The structure contains the *PLCβ3* catalytic core and proximal CTD, two *Gβγ* molecules, and one *Gα_q_* molecule (Fig. 5 and *SI Appendix*, Fig. S6). The CTD linker and distal CTD were disordered, as in the other structures of *PLCβ3*·G protein complexes on membranes (14). The structure is very similar to the structures of *PLCβ3* in complex with each G protein on its own, with no additional conformational changes observed (Fig. 5 *B* and *C*). The X-Y linker is present in the active site and the Hα2’ is in the *Gα_q_*-bound conformation (Fig. 5 *A* and *B*). Each of the *PLCβ3*·G protein interfaces is unaltered by the presence of the additional G protein (Fig. 5 and *SI Appendix*, Fig. S7 *B*–*D* and Tables S2 and S3). These observations are consistent with the functional experiments, which show that binding of one G protein does not influence the other, and that they act independently to give a product rule for catalytic enhancement when both G proteins are present.

### Membrane Association of *PLCβ3*·G Protein Complexes.

**Fig. 6.**
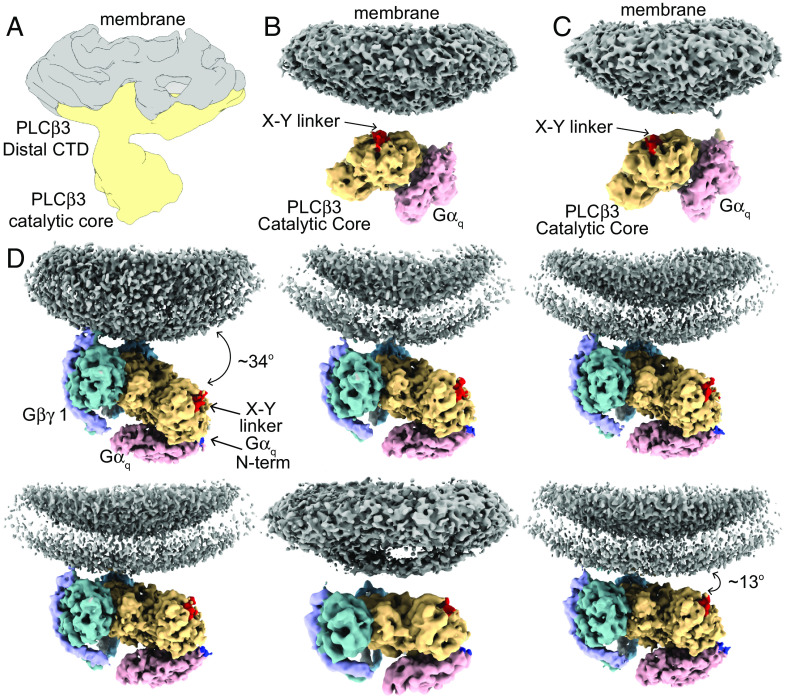
Membrane association of *PLCβ3* in the presence of G proteins. (*A*) Unsharpened reconstruction of *PLCβ3* bound to lipid vesicles in the absence of G proteins shown for comparison (14). *PLCβ3* is colored in yellow and the membrane is colored in gray. (*B* and *C*) 3D reconstructions of two different orientations of the *PLCβ3·Gα_q_* complex on the membrane surface. The reconstructions are colored by protein, *PLCβ3* is yellow, *Gα_q_* is pink, and the membrane is gray. The *PLCβ3* X-Y linker is colored red to highlight the active site within the catalytic core. (*D*) 3D reconstructions of six 3D classes of the *PLCβ3·Gβγ(2)·Gα_q_* complex on membranes showing different positions of the complex with respect to the membrane arranged by degree of tilting. The reconstructions are colored by protein as in *B* and *C*, and *Gβ* 1 is dark teal, *Gγ* 1 is purple, *Gβ* 2 is light blue, *Gγ* 2 is gray. The N terminus of *Gα_q_* is colored blue for reference.

Unmasked classification on the aligned particle subsets for each complex yielded reconstructions with density for the lipid bilayer, allowing us to study the orientation of each complex on the membrane (Fig. 6). Two different membrane-associated reconstructions of the *PLCβ3·Gα_q_* complex were observed, in which the catalytic core associates with the membrane and orients the active site toward the membrane (Fig. 6 *B* and *C*). There were no differences in the protein components of each reconstruction, suggesting that the complex tilts on the membrane as a rigid body (Fig. 6 *B* and *C*). This orientation differs from *PLCβ3* in the absence of G proteins, where the catalytic core extends away from the membrane (Fig. 6*A*) (14). This orientation also differs from the *PLCβ3·Gβγ(2)* complex where the two *Gβγs* anchor the catalytic core to the membrane on the opposing side, resulting in the catalytic site tilting away from the membrane (14).

The *PLCβ3·Gα_q_* orientation seems more poised for catalysis as the active site is oriented directly toward the membrane (Fig. 6 *B* and *C*). It appears likely that this orientation is driven by the *Gα_q_*-induced conformational change of the Hα2’ because, when *Gα_q_* binds, the Hα2’ is displaced from the catalytic core and an underlying hydrophobic patch is exposed on the surface of the catalytic core (*SI Appendix*, Fig. S8 *A* and *B*). The point of membrane association in the complex is very close to this hydrophobic patch, suggesting that it plays a role in positioning the complex on the membrane (*SI Appendix*, Fig. S8 *C* and *D*). If we fit the catalytic core in the absence of G proteins into the density of the *PLCβ3·Gα_q_* complex on the membrane, the Hα2’ protrudes near the membrane density, suggesting that it could hinder membrane association in this configuration. This suggests that *Gα_q_* binding to *PLCβ3*, even without the lipid anchor, could indirectly play a role in orienting the *PLCβ3* catalytic core on the membrane. Such an orientation effect would apply to *PLCβ3s* that have partitioned onto the membrane, rather than on the partitioning step, consistent with the observation that *Gα_q_* does not alter membrane association. It is possible that this orientation of the *PLCβ3·Gα_q_* complex on the membrane contributes to the *Gα_q_*-mediated displacement of the X-Y linker from the active site.

For the complex with both G proteins, the orientation resembles that of the *PLCβ3·Gβγ(2)* complex, where the two *Gβγs* firmly anchor the PH domain and EF hands to the membrane and the other side of the catalytic core tilts away (Fig. 6*D*) (14). We also observed variation in orientation of the complex with *PLCβ3* and both G proteins on the membrane, as in the *PLCβ3·Gβγ(2)* complex. Six reconstructions with at least 4 Å resolution were observed with differing tilt angles of the catalytic core with respect to the membrane, ranging from 34° in the most tilted to 13° in the least tilted (Fig. 6*D*). There are no changes to the protein components in these reconstructions, suggesting again that the complex tilts on the membrane as a rigid body. The membrane orientation seems to be driven by the *Gβγs* under these conditions, which we speculate is due to the lipid anchor on *Gβγ* and the lack thereof on *Gα_q_*.

However, the observed orientations are not incompatible with a lipid anchor on *Gα_q_*, which would likely be present in a cell. In our structures, the N terminus of *Gα_q_* is disordered until position 38 (Fig. 6*D*, blue region) and the lipid modifications are placed on cysteines at positions 9 and 10. Even in our most tilted reconstruction, where the *Gα_q_* N terminus is ~85 Å from the membrane (Fig. 6*D*), the disordered portion is long enough for the lipid anchors to reside in the membrane. This observation is consistent with other structures of Gα subunits in complex with their effectors, including adenylyl cyclase and TRPC5 (27–30), where the Gα is positioned ~50 Å from the membrane and the N terminus is disordered. These observations are consistent with our functional experiments showing that *Gα_q_* activates *PLCβ3* by increasing *k_cat_* rather than through membrane recruitment. However, it is possible that a lipidated *Gα_q_* might also recruit *PLCβ3* to the membrane in addition to increasing its *k_cat_*.

## Discussion

**Fig. 7.**
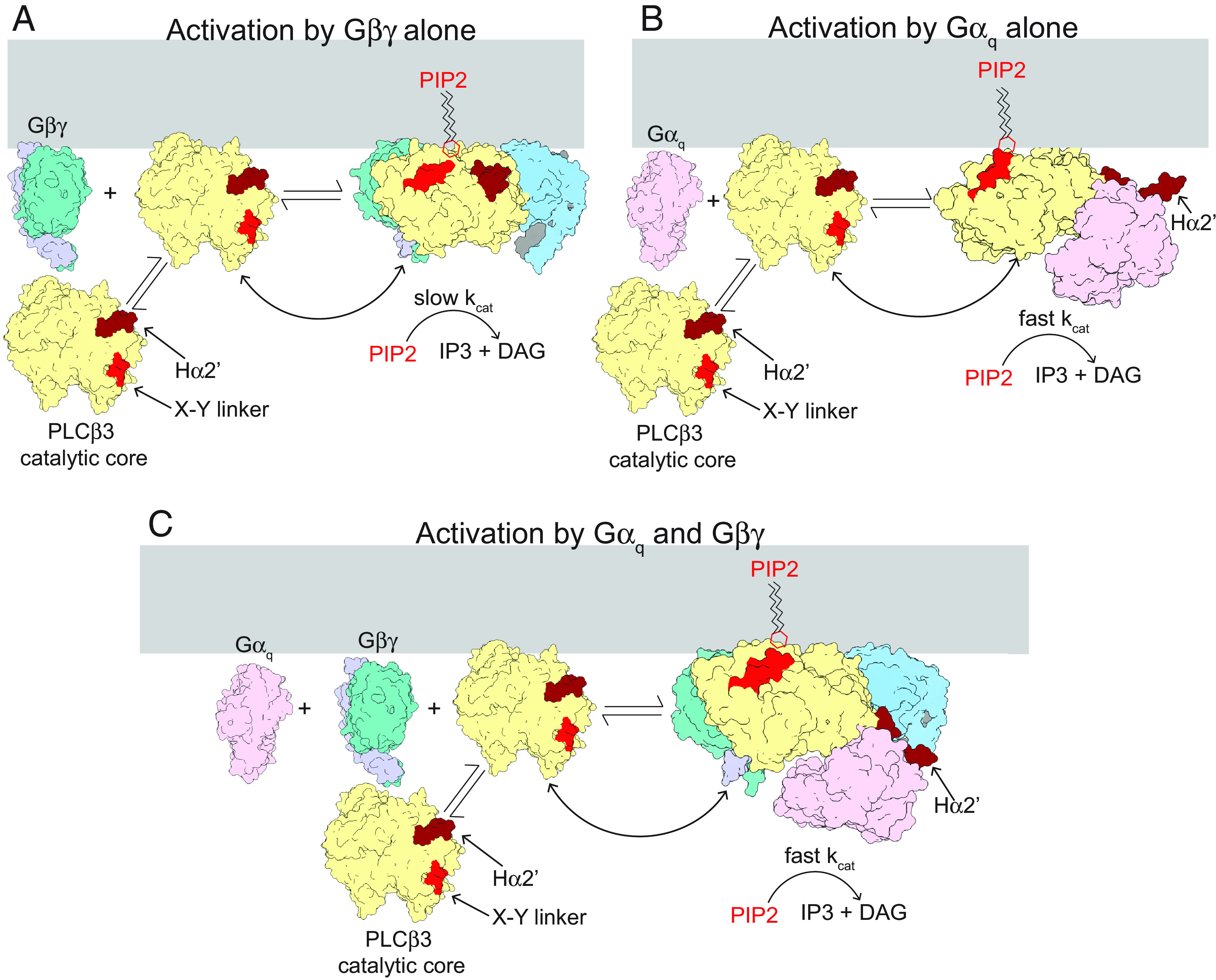
Hypothesized mechanism of activation of *PLCβ* enzymes by *Gβγ* and *Gα_q_*. When *PLCβ3* binds the membrane, the active site is positioned away from the membrane and the enzyme is autoinhibited by both the X-Y linker and the Hα2’, resulting in low activity in the absence of G proteins. (*A*) Free *Gβγ* binds to membrane-associated *PLCβ3*, increases its concentration at the membrane and orients the active site for catalysis, leading to an increase in *PIP2* degradation. However, the *k_cat_* is limited by both the X-Y linker and the Hα2’ (shown in red and dark red, respectively). (*B*) Free *Gα_q_* binds to membrane associated *PLCβ3*, displaces the autoinhibitory Hα2’ (shown in dark red) and the X-Y linker is more frequently absent from the active site, resulting in an increase in *k_cat_* and *PIP2* turnover. (*C*) Free *Gα_q_* and *Gβγ* both bind to membrane-associated *PLCβ3*, leading to a combination of the activation effects of each G protein. The final result is increased *PLCβ3* on the membrane surface with reduced autoinhibition (both the Hα2’ and the X-Y linker) at the membrane, leading to robust *PIP2* hydrolysis. The distal CTD of *PLCβ3* was omitted for clarity.

In a recent study, we analyzed the structural and enzymatic properties of *PLCβ3* in the absence and presence of *Gβγ* on lipid vesicles (14). We found that *PLCβ3* catalyzes *PIP2* hydrolysis in accordance with Michaelis-Menten enzyme kinetics with a very small *k_cat_* (~1.7 *s*^*−1*^) but that *Gβγ* can increase net catalysis by binding to *PLCβ3* and thus recruiting it to the membrane. It is known that *Gα_q_* also increases net catalysis (8, 10, 11, 16). In this study, we investigate the influence of *Gα_q_* on *PLCβ3* activity. We used *Gα_q_* that does not contain a lipid anchor. Our essential findings are as follows: 1) Nonlipidated *Gα_q_* increases *V_max_* in a concentration-dependent manner, following a rectangular hyperbola, consistent with 1:1 binding of *Gα_q_* to *PLCβ3*. The apparent equilibrium constant for binding is ~120 *nM*, and maximal activation is ~35-fold greater than the basal (i.e., in the absence of *Gα_q_*) catalytic rate. 2) *Gα_q_* without a lipid anchor does not partition onto the membrane surface nor does it influence the degree to which *PLCβ3* partitions onto the membrane surface. Thus, *Gα_q_* without a covalent lipid anchor increases *V_max_* by increasing *k_cat_*. 3) The ability of *Gα_q_* to increase *k_cat_* depends on the presence of the X-Y linker autoinhibitory element on *PLCβ3*. 4) *Gα_q_* and *Gβγ* act independently to increase *V_max_*. Consequently, when both G proteins are applied simultaneously, the net increase in *PLCβ3* catalytic activity is given by the product of the two individual effects. Under the conditions in which we have studied *PLCβ3* enzyme activity, maximal dual stimulation can increase *PIP2* hydrolysis greater than 2,000-fold. 5) Structures of *PLCβ3* on lipid membrane vesicles alone, with *Gα_q_*, with *Gβγ*, and with both G proteins together, show that two *Gβγ* and one *Gα_q_* bind to *PLCβ3* simultaneously and independently, consistent with their influence on *PLCβ3* catalysis. In summary, two *Gβγ* localize (i.e., recruit) *PLCβ3* to the membrane. Independently, *Gα_q_* increases *k_cat_*. Mutational studies support the hypothesis that *Gα_q_* regulates *k_cat_* allosterically through the autoinhibitory X-Y linker (Fig. 7).

There is one difference in the conditions of our partitioning experiments and the kinetic experiments for *PLCβ3* function: The partitioning experiments are carried out in the absence of *PIP2*. We could not include *PIP2* in the partitioning experiments because it would be hydrolyzed throughout the measurement. However, if *PIP2* did influence the partition coefficient for *PLCβ*3, it would not affect our conclusion that *Gα_q_* (without a lipid anchor) does not alter the local concentration *PLCβ*3 in the membrane and thus increases *V_max_* by increasing *k_cat_*. As shown in Fig. 2*D*, *Gα_q_* does not alter the fraction of *PLCβ*3 partitioned, whereas *Gβγ* does. Enhanced partitioning caused by *Gβγ* accounts for most of its effect on catalysis (14). That *Gα_q_* does not enhance partitioning is independent of the precise value of the *PLCβ*3 partition coefficient. Thus, we can attribute the ability of *Gα_q_* to increase the *V_max_* of *PLCβ3* by ~35-fold as an increase in *k_cat_*, not its local concentration.

In the enzyme assay, *k_cat_* for *PLCβ3* without *Gα_q_* stimulation is ~1.7 *s*^*−1*^ (14), with maximal *Gα_q_* stimulation ~60 *s*^*−1*^, and with the X-Y linker removed by mutation ~2,000 *s*^*−1*^. If we take 2,000 *s*^*−1*^ as the magnitude of *k_cat_* without autoinhibition, then wild-type *PLCβ3* in the absence of *Gα_q_* is inhibited by the X-Y linker more than 99.9% of the time and in the presence of a maximally activating concentration of *Gα_q_* it is still inhibited about 97% of the time. On top of this, the partition coefficient of *PLCβ3* is such that nearly all of it in a cell is in the aqueous solution, not on the membrane, in the absence of G protein stimulation (14). Why has nature so severely suppressed the catalytic activity of this enzyme? The answer, we propose, is that excessive background activity of *PLCβ3* activity will have severe consequences for the stability of cells. In fact, naturally occurring mutations show this to be the case (31–33). Not only does *PIP2* regulate the activity of many membrane channels, transporters and receptors, but of equal importance, the products of *PLCβ3*-mediated *PIP2* hydrolysis, *DAG* and *IP3*, regulate protein kinase C and the *IP3* receptor, which control phosphorylation of many proteins and intracellular Ca^2+^ concentration, respectively. Therefore, we propose that there is strong evolutionary “pressure” to minimize baseline *PLCβ3* activity. Combining the results in our previous study (14) and in the present study, we can understand how, in the setting of intense catalytic suppression, catalysis still occurs in abundance when it is called for (Fig. 7). *Gβγ*, by binding to *PLCβ3*, recruits it to the membrane (Fig. 7*A*). Simultaneously, *Gα_q_* can increase *k_cat_* ~35 fold through partial relief of X-Y linker inhibition (Fig. 7*B*). We show that under the conditions of our experiments, together these two regulatory mechanisms can enact a greater than 2,000-fold increase in *PLCβ3* activity (Fig. 7*C*). Lipidated *Gα_q_* may further concentrate *PLCβ3* on the membrane, leading to an even greater increase in activity upon receptor stimulation.

While our experiments leave little question about the involvement of the X-Y linker in *Gα_q_*-dependent activation, it remains unclear exactly how *Gα_q_* binding alters the association of the linker in the active site. The only observed conformational change in the protein upon *Gα_q_* binding is the displacement of the Hα2’ away from the catalytic core (Fig. 4*D*). Perhaps the displacement of this helix increases the dynamics in the catalytic core, allowing the X-Y linker to be displaced more frequently as previously proposed (34, 35). We also observed a *Gα_q_*-dependent change in orientation of the catalytic core on the membrane, which could be related to the Hα2’ displacement (Fig. 6 *B* and *C* and *SI Appendix*, Fig. S8). This change in membrane orientation is consistent with previous results showing that the membrane plays a role in Hα2’ autoinhibition and that *Gα_q_* only activates *PLCβ3* in the presence of membranes (13). In the *Gα_q_*-dependent orientation, the *PLCβ3* active site is oriented toward the membrane, which could potentially displace the X-Y linker through repulsion of its adjacent acidic stretch by the negatively charged lipids. Such a mechanism has been previously proposed (8, 13), but our observations offer a new subtlety in that the linker could be transiently displaced based on the orientation of the catalytic core on the membrane rather than a stable displacement following membrane partitioning. Involvement of the Hα2’, as in either of these potential mechanisms, leads to the proposal that the autoinhibitory function of the Hα2’ is related to its coupling to the X-Y linker. However, previous studies have proposed that the autoinhibition by the Hα2’ and the X-Y linker are independent (9). Further experiments are necessary to fully understand the mechanism of X-Y linker displacement by *Gα_q_* and Hα2’ autoinhibition.

As described above, the results from our reconstitution experiments have many implications for signaling in the cellular environment. For example, the observed affinity of *PLCβ3* for *Gα_q_* is relatively high, suggesting that a low level of receptor stimulation can lead to robust *PLCβ3* signaling. This effect would be further amplified in the cellular context with lipidated *Gα_q_*, which might also increase the local concentration of *PLCβ3* on the membrane. Furthermore, because *Gβγ* and *Gα_q_* activate *PLCβ3* by different mechanisms and coactivate as the product of the two influences of each G protein, *PLCβ3* is well poised to serve as a coincidence detector of costimulation by *Gα*_*i*_ and *Gα_q_* coupled receptors, even under low levels of costimulation, which would be important for many physiological processes (8, 17, 18).

## Materials and Methods

### Protein Expression, Purification, and Reconstitution.

All proteins were purified according to previously established protocols using affinity chromatography and size exclusion chromatography. Detailed methods are described in *SI Appendix, Materials and Methods: Protein Expression and Purification and Protein Reconstitution*.

### *PLCβ3* Functional Assay.

*PLCβ3* activity was measured using a planar lipid bilayer setup and a *PIP2*-dependent ion channel to report *PIP2* concentration in the membrane over time. Detailed methods are described in *SI Appendix, Materials and Methods: Bilayer Experiments and Analysis*.

### Membrane Partitioning Experiments.

*Gα_q_* or fluorescently labeled *PLCβ3* was mixed with LUVs and pelleted. Protein in the pellet and supernatant was quantified using fluorescence. Detailed methods are described in *SI Appendix, Materials and Methods: PLCβ3 and Gα_q_ Vesicle Partition Experiments*.

### *PLCβ3·G* Protein Complex Structure Determination.

*PLCβ3·Gα_q_* complex was mixed with liposomes with or without *Gβγ* prior to sample vitrification. Cryo-EM data were collected using a Titan Krios with a Gatan K3 direct detector according to the parameter values in *SI Appendix*, Table S1 and analyzed according to the procedures outlined in *SI Appendix*, Figs. S5 and S6. Atomic models from previously determined structures were fit into our density maps, refined using PHENIX real-space refine (36), and manually adjusted. Detailed methods are described in *SI Appendix, Materials and Methods: Cryo-EM Sample Preparation and Data Collection, Cryo-EM Data Processing, and Model Building and Validation*.

## Supplementary Material

Appendix 01 (PDF)

## Data Availability

Cryo-EM maps and atomic models for all structures described in this work have been deposited to the Electron Microscopy Data Bank (EMDB) (*PLCβ·Gα_q_*: EMDB-42475 (37) and *PLCβ·Gβγ·Gα_q_*: EMD-42476 (38)) and the Protein Data Bank (PDB) (*PLCβ·Gα_q_*: 8UQN (39) and *PLCβ·Gβγ·Gα_q_*: 8UQO (40)), respectively.
