# Supplementary material for: The mechanism of Gαq regulation of PLCβ3-catalyzed PIP2 hydrolysis
Source: Proc Natl Acad Sci U S A. 2023 Nov 22;120(48):e2315011120. doi: 10.1073/pnas.2315011120 (PMC10691244; doi:10.1073/pnas.2315011120)
Supplement: Supplementary file 1 — Appendix 01 (PDF) [file pnas.2315011120.sapp.pdf]

PNAS

Supporting Information for

The mechanism of $G\alpha_q$ regulation of *PLCβ3*-catalyzed *PIP2* hydrolysis.

Maria E. Falzone[1,2] and Roderick MacKinnon[1,2*]

[1]Laboratory of Molecular Neurobiology and Biophysics, The Rockefeller University, New York, United States. [2]Howard Hughes Medical Institute, The Rockefeller University, New York, United States.

*Roderick MacKinnon

Email: mackinn@rockefeller.edu

This PDF file includes:

Figures S1 to S8
Tables S1 to S3
SI Materials and Methods
Appendix 1
Appendix 2
SI References

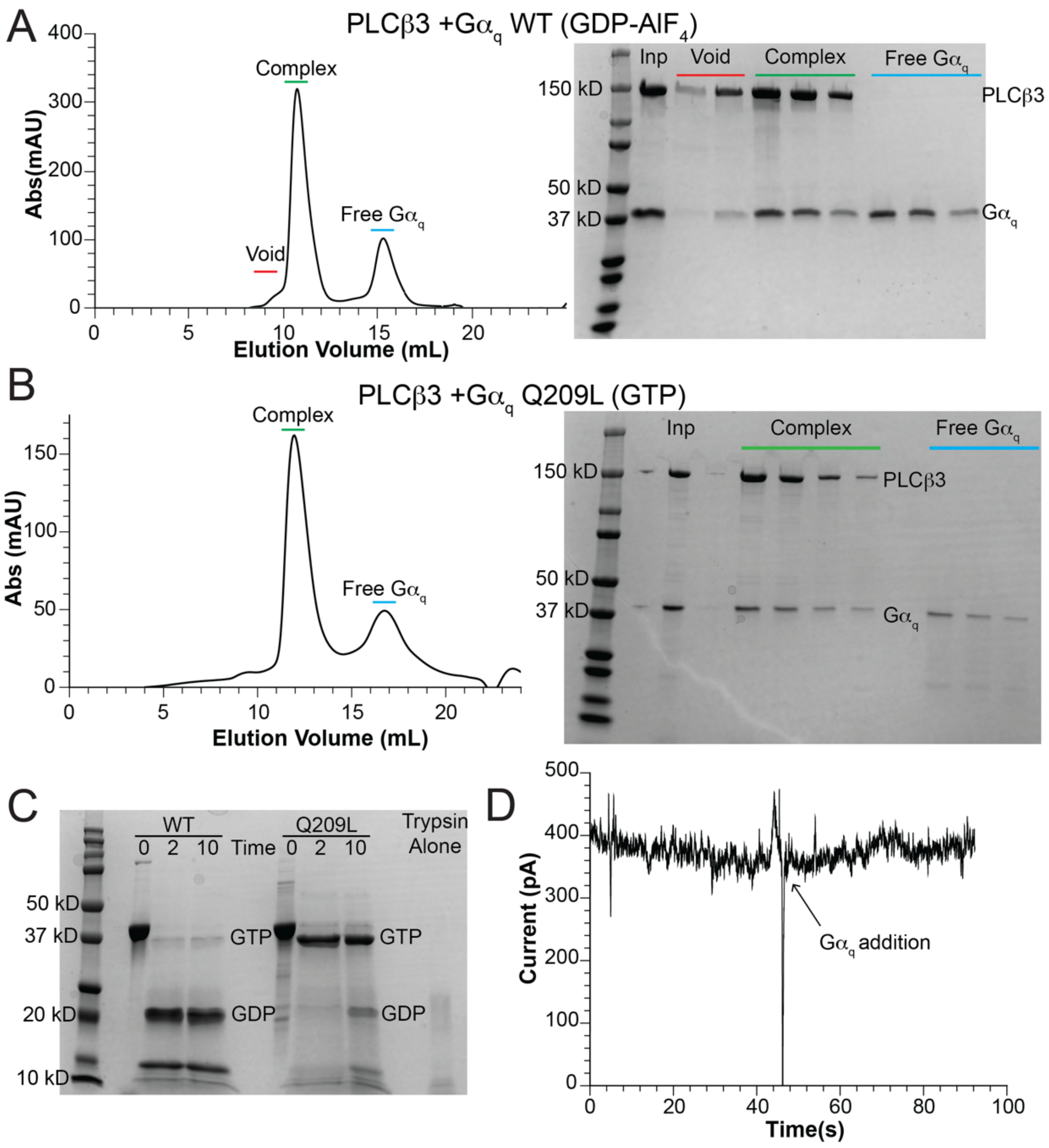

Figure S1: Comparison of $G\alpha_q$ wildtype and hydrolysis deficient mutant, Q209L. A-B: *PLCβ3*·$G\alpha_q$ complex formation with wildtype $G\alpha_q$ in the presence of GDP-AlF_4 (A) or $G\alpha_q$ Q209L in the presence of GTP (B) with size exclusion chromatography profiles of the complex on a Superdex 200 10/300 increase column are on the left and accompanying SDS-PAGE gels are on right. In both cases, *PLCβ3* was mixed with a two-fold molar excess of $G\alpha_q$, and $G\alpha_q$ comigrates with *PLCβ3*. Inp refers to the input sample prior to size exclusion chromatography. C: SDS-PAGE gel of trypsin cleavage assay (1) (see methods) to evaluate the nucleotide state of purified $G\alpha_q$ wildtype (left) or Q209L (right). The time is in minutes and the 0 timepoint is prior to the addition of trypsin. Cleavage of GDP-bound $G\alpha_q$ produces a band ~ 20 kD and cleavage of GTP-bound $G\alpha_q$ produces a band ~37 kD (1). Purification of $G\alpha_q$ Q209L in the presence of GTP yields predominantly GTP-bound protein. D. GIRK current over time before and after addition of 50 *nM* $G\alpha_q$ Q209L GTP showing the current is not affected.

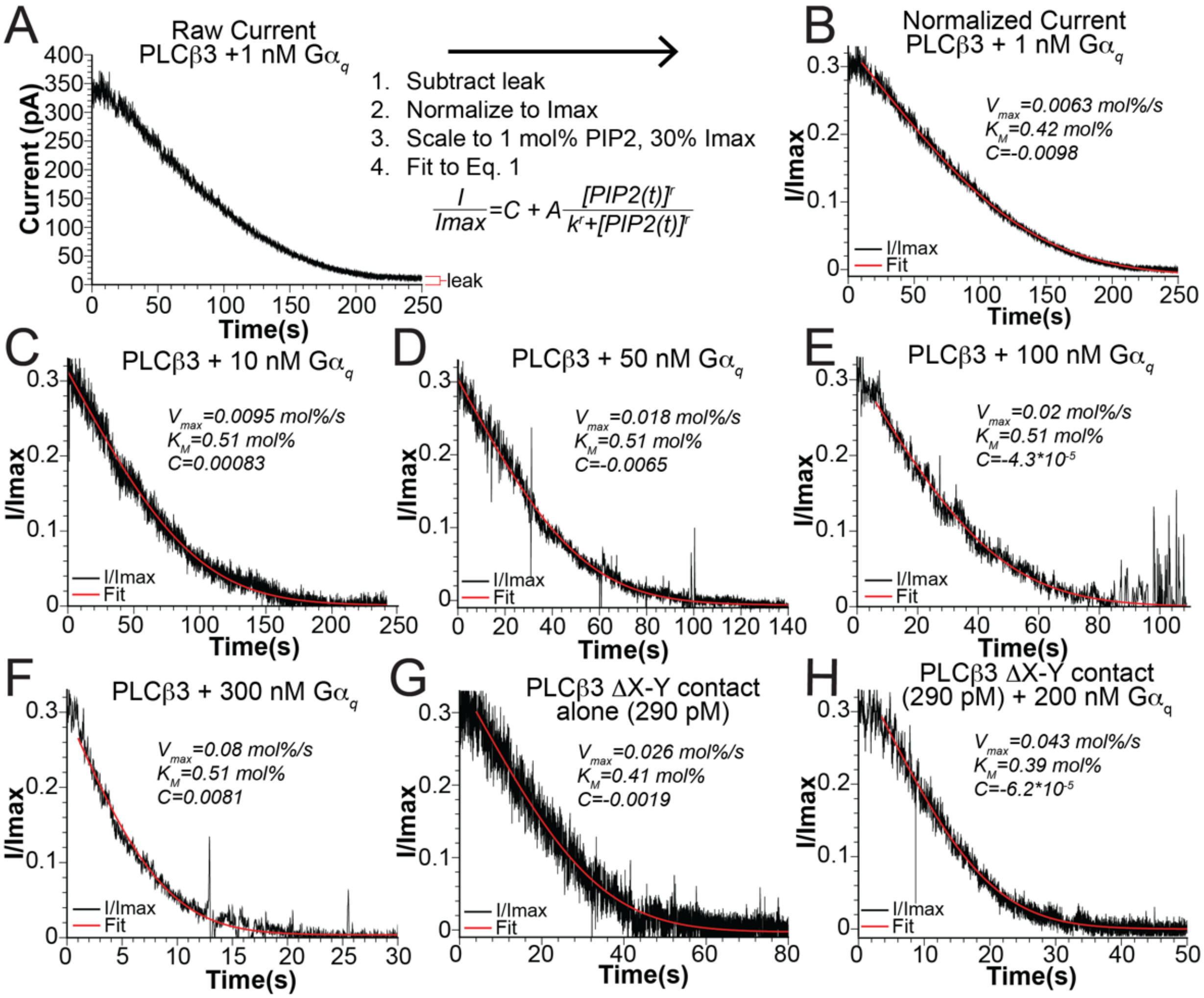

$$\frac{I}{Imax} = C + A\frac{[PIP2(t)]^r}{k^r + [PIP2(t)]^r}$$

Figure S2: *$G\alpha_q$*-dependent activation of *PLCβ3*. A: Raw *PLCβ3·$G\alpha_q$*-induced current decay and analysis scheme. The example shown is in the presence of 29 *nM PLCβ3* and 1.0 *nM $G\alpha_q$*. The red bars in the bottom right corner of the trace demarcate the background leak current that remains in the bilayer when the current decay is complete. This is the background leak current that is subtracted prior to normalization and further analysis. To determine values for V_{max} and K_M, raw currents were leak subtracted, normalized to the maximum current before the decay, I_{max}, and scaled to the starting level of *PIP2* in the bilayer, 1.0 *mol%*, which is 30% of the maximal GIRK-ALFA current with saturating *PIP2*. The normalized decays were fit to SI Appendix, Eq. S3 with free parameters V_{max}, K_M, and *C*. B-F: Representative normalized current decay (using 29 *nM* of wildtype *PLCβ3*) fit to SI Appendix, Eq. S3 to determine V_{max} and K_M (red curves) in the presence of varying concentrations of $G\alpha_q$, 1.0 *nM* (B), 10 *nM* (C), 50 *nM* (D), 100 *nM* (E), 300 *nM* (F). G-H: Representative normalized current for *PLCβ3* with the structured part of the X-Y linker deleted (ΔX-Y contact) using 290 *pM* enzyme fit to SI Appendix, Eq. S3 to determine V_{max} and K_M (red curves) in the absence (H) and presence (I) of 200 *nM $G\alpha_q$*.

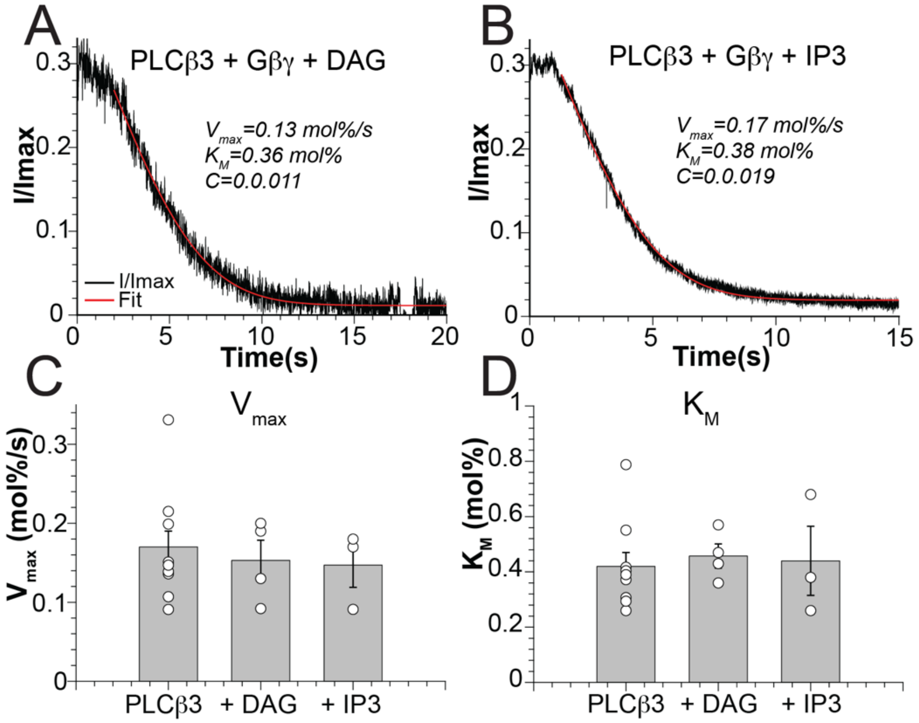

Figure S3: *PLCβ3* is not inhibited by the products of its reaction, *IP3* and *DAG*, in our experimental setup. A-B: normalized *PLCβ3*-dependent current decays using 29 *nM* enzyme in the presence of lipidated *Gβγ* and 1.0 *mol% DAG* (A) or 1.0 *μM IP3* (B). Red curves are fits to SI Appendix, Eq. S3. to determine V_{max} and K_M. R^2=0.97 (A) and 1.0 (B). C-D: comparison of V_{max} (C) or K_M (D) in the presence and absence of 1.0 *mol% DAG* and 1.0 *μM IP3*. The bars are the mean, the error bars are standard error of mean, and the circles are values from individual experiments. Data for *PLCβ3* alone were reported in (2).

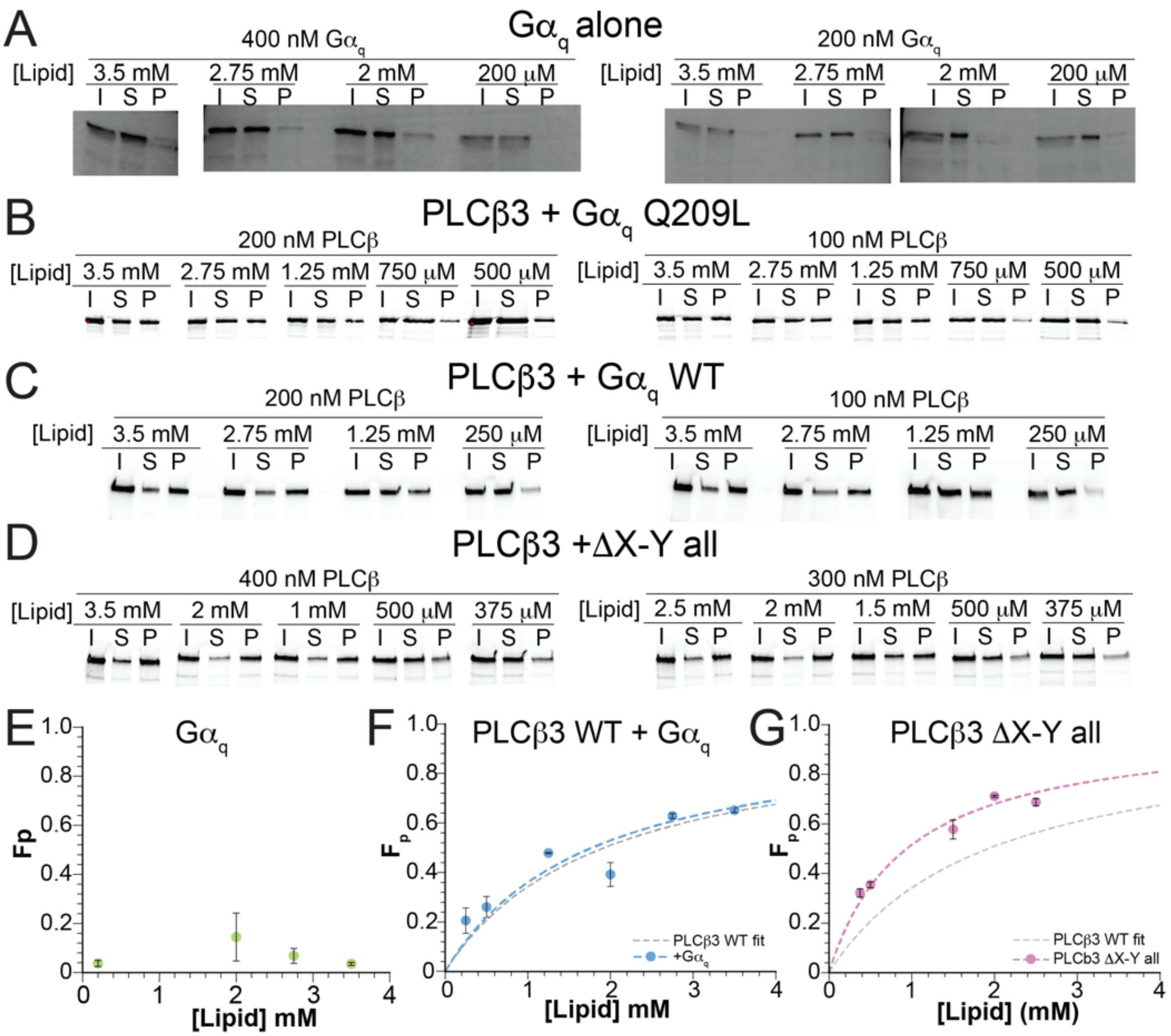

Figure S4: *PLCβ3* partitioning in the presence of $G\alpha_q$. A: Silver stained SDS-PAGE gels of partitioning experiments for 200 or 400 *nM* wildtype $G\alpha_q$ with GDP-AlF4 (plotted in E). I is input, S is supernatant, and P is pellet. No membrane partitioning behavior is evident as protein in the pellet (P) does not increase with increasing lipid concentration. B-D: SDS-PAGE gels imaged for LD655 fluorescence of 100 or 200 *nM* wildtype *PLCβ3* partitioning experiments in the presence of 200 *nM* $G\alpha_q$ Q209L with GTP (B), 200 *nM* wildtype $G\alpha_q$ GDP-AlF4 (C) or 400 and 300 *nM* *PLCβ3* ΔX-Y all (D). E-G: Membrane partitioning curve for $G\alpha_q$ alone (E), wildtype *PLCβ3* in the presence of wildtype $G\alpha_q$ with GDP-AlF4 (F-blue) or *PLCβ3* ΔX-Y all (G-pink) for 2DOPE:1POPC:1POPS LUVs with Fraction Partitioned (F_p) on the Y axis. Points are the average from 2 repeats for each lipid concentration and error bars are range of mean. Points in E and F were fit to Eq. 5 to determine K_x (dashed blue or pink curve). For wildtype *PLCβ3* in the presence of wildtype $G\alpha_q$, K_x=3.1*10^4, R^2=0.81. For *PLCβ3* ΔX-Y all, K_x=5.8*10^4, R^2=0.96. The fit to Eq. 5 for wildtype *PLCβ3* alone is shown as a gray dashed curve for reference (2).

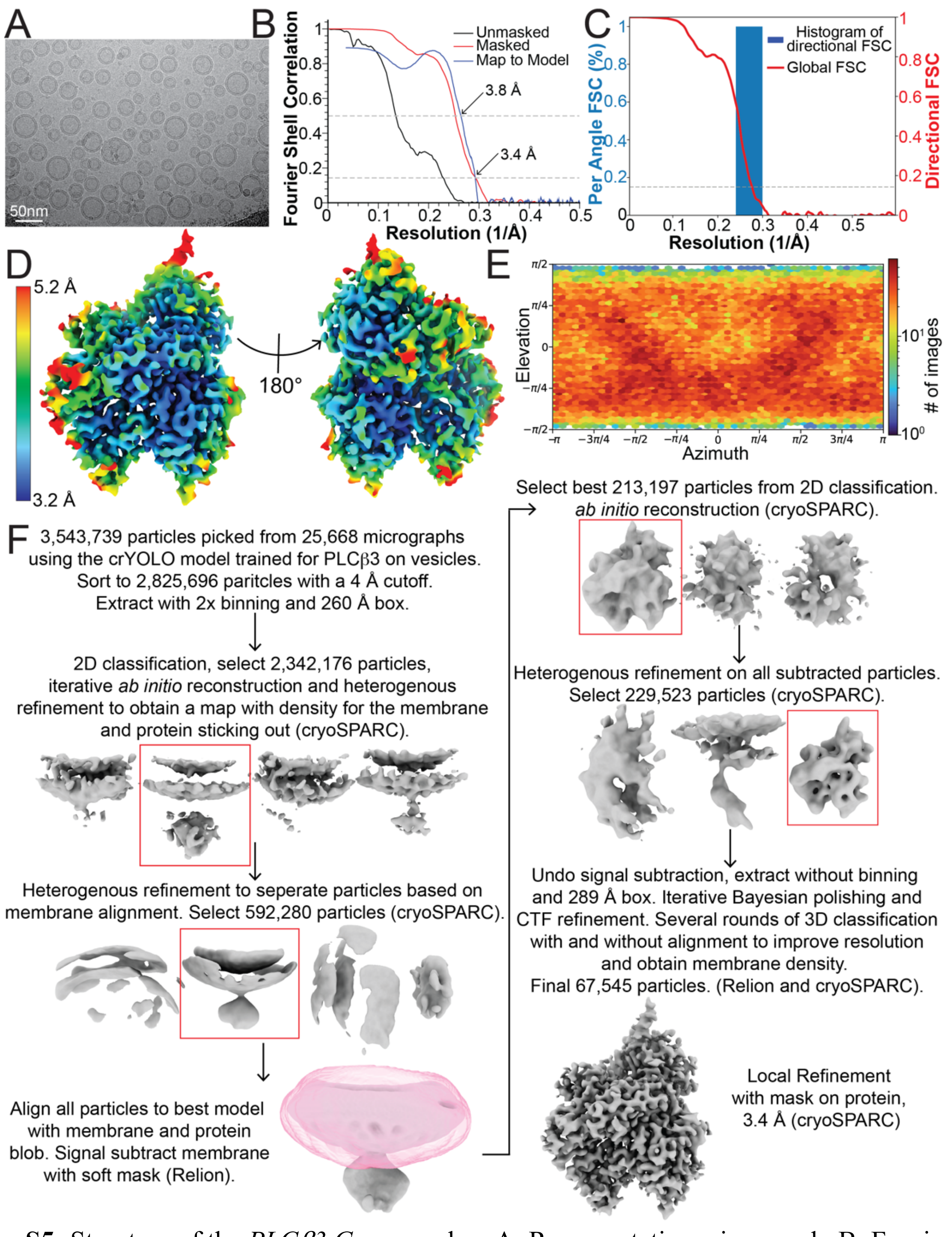

Figure S5: Structure of the $PLC\beta3{\cdot}G\alpha_q$ complex. A: Representative micrograph. B: Fourier shell correlation (FSC) curves for the unmasked (black) and masked (red) maps and between the map and model (blue). The 0.143 and 0.5 thresholds are denoted by dashed lines. C: 3D FSC plot for the final masked map (3). D: Final masked, sharpened map colored by local resolution determined by cryoSPARC. E: Angular distribution plot for the final masked map from cryoSPARC. F: Summary of data processing steps, see methods. Maps shown are unsharpened.

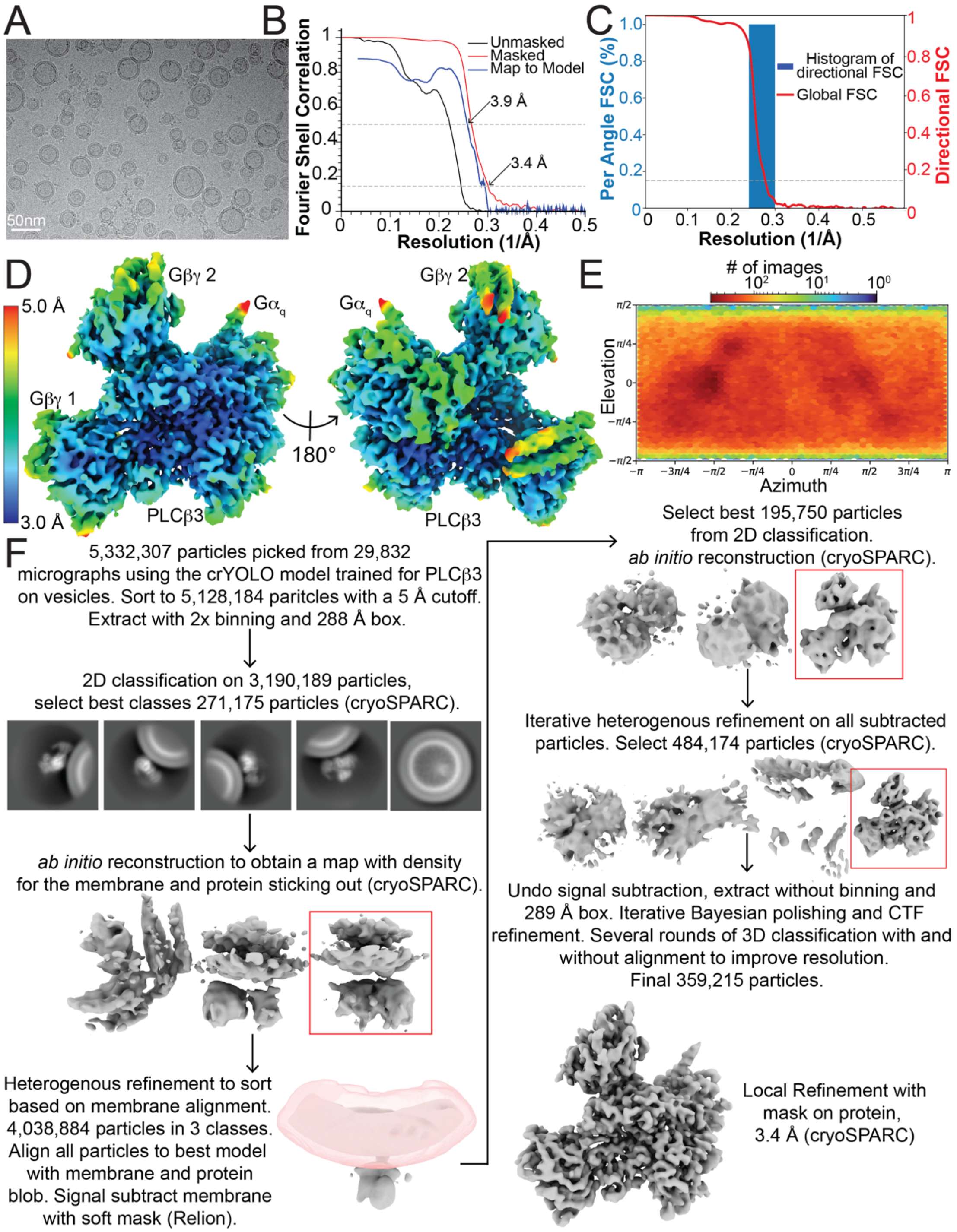

Figure S6: Structure of the *PLCβ3·Gβγ(2)·G*α_q complex. A: Representative micrograph. B: Fourier shell correlation (FSC) curves for the unmasked (black) and masked (red) maps and between the map and model (blue). The 0.143 and 0.5 thresholds are denoted by dashed lines. C: 3D FSC plot for the final masked map (3). D: Final masked, sharpened map colored by local resolution determined by cryoSPARC. E: Angular distribution plot for the final masked map from cryoSPARC. F: Summary of data processing steps, see methods. Maps shown are unsharpened.

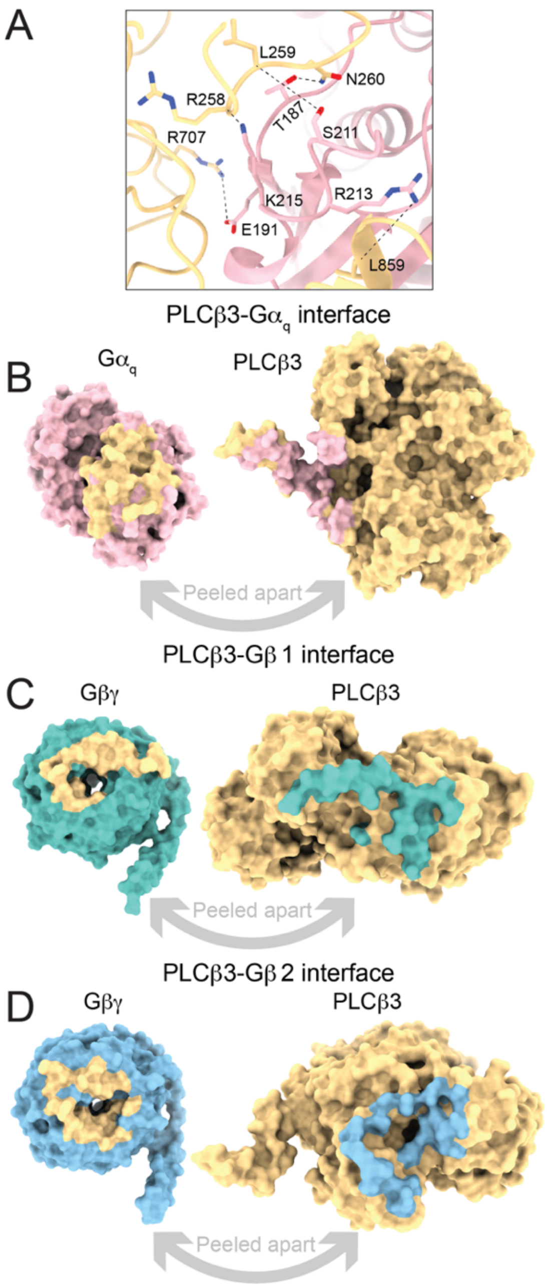

Figure S7: *PLCβ3*-G protein interfaces. A: Hydrogen bonds at the *PLCβ3·G*α_q interface. *PLCβ3* is yellow and *G*α_q is pink. Relevant side chains are shown as sticks and colored by heteroatom. Hydrogen bonds are denoted by dashed black lines. All hydrogen bonds shown are less than 3.5 Å apart. B-D: Surface representation of the *PLC-G*α_q (B), *PLCβ3-Gβ* 1 (C), or *PLCβ3-Gβ* 2 (D) interfaces in the *PLCβ3·Gβγ(2)·G*α_q complex peeled apart to show extensive interactions. *PLCβ3* is yellow, *G*α_q is pink, *Gβ* 1 is dark teal, and *Gβ* 2 is light blue. *Gγ* 1 and 2 were omitted for clarity. Residues on *PLCβ3* that interact with G proteins are colored according to the corresponding G protein and residues on the G proteins that interact with *PLCβ3* are colored in yellow. Interface residues were determined using the ChimeraX interface feature using a buried surface area cutoff of 15 Å^2. Interfaces are comparable to structures determined with each G protein on its own.

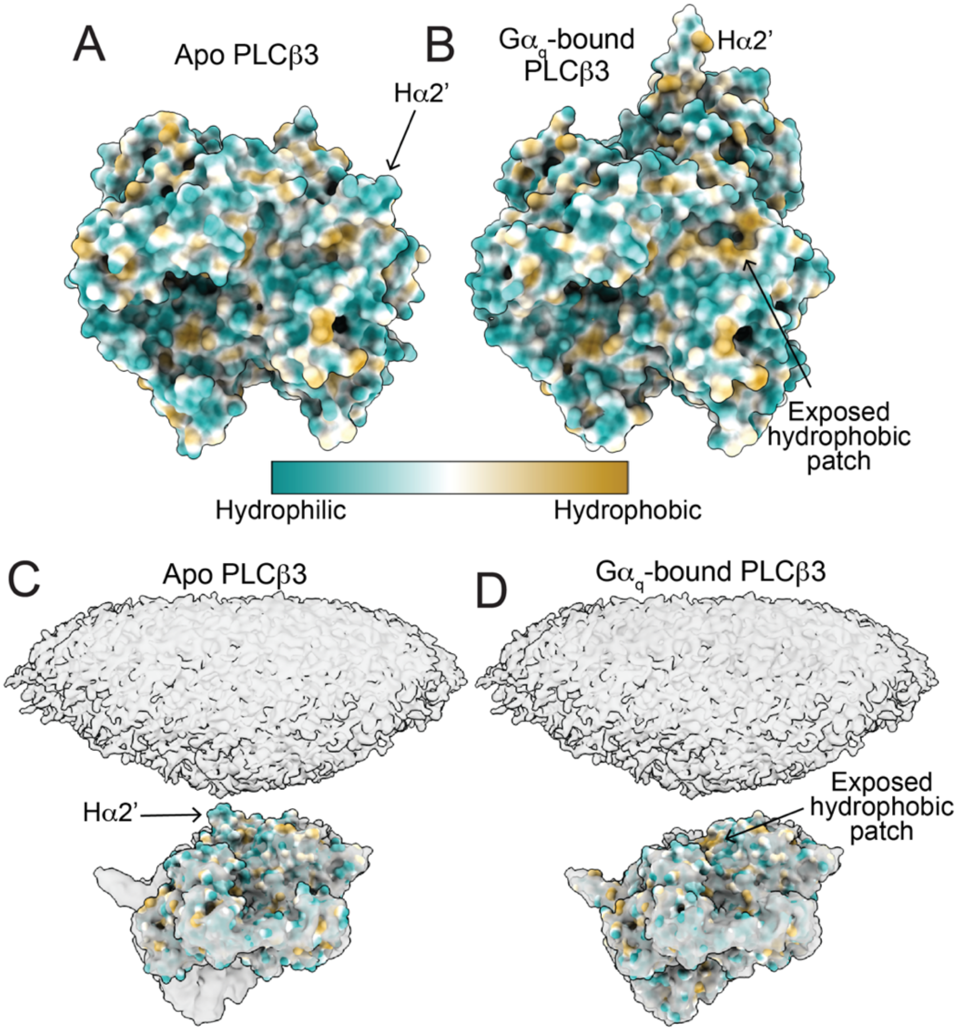

Figure S8: Potential role of the Hα2' in membrane association of the *PLCβ3* catalytic core. A-B: Catalytic core of apo (A) or $G\alpha_q$-bound (B) *PLCβ3* colored by hydrophobicity highlighting a hydrophobic patch covered by Hα2' in the apo conformation. C-D: Hydrophobicity-colored apo (C) or $G\alpha_q$-bound (D) *PLCβ3* catalytic core fit into the density of the membrane associated $PLC\beta3{\cdot}G\alpha_q$ complex. The exposed hydrophobic patch is close to the position of membrane association in the $G\alpha_q$-bound conformation and the Hα2' associated with the catalytic core could hinder membrane association.

Table S1: Cryo-EM collection parameters and model statistics

Collection Parameters	*PLCβ3·Gα$_q$* complex	*PLCβ3·Gβγ(2)·Gα$_q$* complex
Accelerating Voltage (kV)	300	300
Number of frames	50	50
Dose ($e^-/Å^2$)	60	60
Defocus Range (μm)	1.5-2.5	1.5-2.5
Exposure Time (s)	4.26	3.86152
Original Pixel size (Å)	0.4195	0.4195
Map Parameters		
Final Pixel Size (Å)	0.839	0.839
Symmetry	C1	C1
Total micrographs	25,668	29,832
Initial particles	3,543,739	5,332,307
Final particles	67,545	359,215
Map resolution (Å)	3.43	3.37
FSC threshold	0.143	0.143
Map local resolution range (Å)	3.2-5.2	3-5
Map sharpening B factor ($Å^2$)	-119.5	-137
Initial Model Used	4GNK	8EMW
Model resolution (Å) (FSC_{model}=0.5)	3.8	3.9
Model Composition		
Nonhydrogen atoms	8,553	13,960
Protein residues	1069	1823
Ligands	4	4
r.m.s. deviations bond length (Å)	0.008	0.011
r.m.s. deviations bond angles (°)	0.804	1.092
Validation		
MolProbity Score	1.71	1.99
Clash Score	6.67	9.24
Poor Rotamers (%)	0	0.07
Ramachandran Plot		
Favored (%)	95.0	91.68
Allowed (%)	5.0	8.32
Disallowed (%)	0	0

Table S2: *PLCβ3*·G protein interface buried area comparison.

Complex	*Gβγ 1 interface (Å²)*	*Gβγ 2 interface (Å²)*	*Gα_q interface (Å²)*
PLCβ3·Gβγ[1]	807	1105	-
PLCβ3·Gα_q	-	-	1467
PLCβ3·Gβγ(2)·Gα_q	827	1001	1482

1. Previously reported (2)

Table S3: *PLCβ3*·G protein interface residue comparison. Interface residues were determined using the ChimeraX interface feature using a buried surface area cutoff of 15 $Å^2$.

Complex	*Gβγ 1 interface*	*Gβγ 2 interface*	*Gα_q interface*
PLCβ3·Gβγ[1]	34	44	-
PLCβ3·Gα_q	-	-	56
PLCβ3·Gβγ(2)·Gα_q	35	44	56

1. Previously Reported (2)

SI Materials and Methods

Protein Expression and Purification

Protein expression and purification was caried out as previously described (2). Following expression of all proteins, cell pellets were flash frozen and stored at -80°C until use. All protein purification steps were carried out at 4°C. Protease inhibitors (12.5 *μg/μL* leupeptin, 12.5 *μg/μL* pepstatin A, 625 *μg/μL* AEBSF, 1 *mM* Benzamadine, 100 *μg/μL* Trypisin inhibitor, 1x aprotinin, and 1 *mM* PMSF) and dNase were added to all cell lysates. GFP nanobody-coupled Sepharose resin used for protein purification was prepared as previously described (2).

The *PLCβ3* construct was comprised of human *PLCβ3* residues 10-1234 (provided by Dr. Sondek (4), downstream of GFP with a 3C protease cleavage site between the GFP and *PLCβ3*. For the X-Y linker deletion constructs, deleted residues (471-584 for ΔX-Y all, and 575-585 for ΔX-Y contact) were replaced with a 12 amino acid GSSG linker using the KLD enzyme mix (NEB). Expression and purification were carried out the same as for the wildtype protein. High Five insect cells were infected with 15-20 *mL* P3 virus per liter and harvested after 36-48 hours by centrifugation at 3,500 x *g* for 15 minutes. Protease inhibitors were used in all buffers, except the *PLCβ3* column wash buffer in which leupeptin, pepstatin A and PMSF were excluded. Cells were resuspended in *PLCβ3* lysis buffer (50 *mM* HEPES pH 8.0, 50 *mM* NaCl, 10 *mM* 2-mercaptoethanol, 5% glycerol (v/v), 0.1 *mM* EDTA, and 0.1 *mM* EGTA) and lysed by brief sonication. Lysate was clarified by centrifugation at 39,000 x *g* for 45 minutes and the supernatant was bound to GFP nanobody-coupled Sepharose resin for one hour. The resin was washed in batch with 10 column volumes of *PLCβ3* column wash buffer (20 *mM* HEPES pH 8.0, 400 *mM* NaCl, 10 *mM* 2-mercaptoethanol, 2% glycerol (v/v), 0.1 *mM* EDTA, and 0.1 *mM* EGTA), then loaded onto a column and washed with an additional 10 column volumes by gravity flow. Protein was eluted by cleavage with 3C PreScission protease for 1.5 hours and concentrated to ~10 *mg/mL* using a 15-*mL* Amicon concentrator with 100-kD molecular weight cutoff. Concentrated protein was subjected to size exclusion chromatography using a Superdex 200 10/300 increase column equilibrated with *PLCβ3* SEC buffer (20 *mM* HEPES pH 8.0, 100 *mM* NaCl, 5 *mM* Dithiothreitol (DTT), 2% glycerol (v/v), 0.1 *mM* EDTA, and 0.1 *mM* EGTA). Fractions containing *PLCβ3* were pooled, flash frozen, and stored at -80°C for later use.

To purify *PLCβ3* for non-specific cysteine labeling, the 2-mercaptoethanol was replaced with 2 *mM* tris(2-carboxyethyl)phosphine (TCEP) and peptide-based protease inhibitors were excluded from the labeling buffer. The protein-loaded resin was washed with *PLCβ3* labeling buffer (20 *mM* HEPES pH 7.4, 400 *mM* NaCl, 2 *mM* TCEP, 2% glycerol (v/v), 0.1 *mM* EDTA, and 0.1 *mM* EGTA) prior to 3C PreScission protease cleavage. Following elution, maleimide LD655 (5) was added in 5-fold molar excess and incubated overnight, protected from light. Labeled protein was concentrated to ~10 *mg/mL* using a 15-*mL* Amicon concentrator with 100-kD molecular weight cutoff and subjected to size exclusion chromatography using a Superdex 200 10/300 increase column in *PLCβ3* SEC buffer. Fractions with labeled *PLCβ3* were pooled and the labeling efficiency was evaluated. Aliquots were flash frozen and stored at -80°C for later use. Labeling efficiency was consistently 30-40%.

For lipidated *Gβγ,* untagged human *Gβ*1 was co-expressed with human *Gγ*2 with an N-terminal His-YFP tag in High Five insect cells by infection with 12 and 8 *mL* of P3 baculovirus, respectively. Cells were harvested 36-48 hours after infection by centrifugation at 3,500 x *g* for

15 minutes. Pellets were resuspended in *Gβγ* lysis buffer (25 *mM* Tris-HCl pH 8.0, and 125 *mM* NaCl) supplemented with 5 *mM* EGTA and 5 *mM* DTT and lysed by manual homogenization. Membranes were collected by centrifugation at 39,000 x *g* for 30 minutes, resuspended in fresh *Gβγ* lysis buffer supplemented with dNase and protease inhibitors and manually homogenized again. *Gβγ* was extracted using 1% sodium cholate for 1.5 hours and centrifuged at 39,000 x *g* for 30 minutes. The supernatant was bound in batch to TALON resin equilibrated with *Gβγ* lysis buffer supplemented with 1% sodium cholate for one hour. The resin was washed in batch with 10 column volumes of *Gβγ* lysis buffer supplemented with 1% sodium cholate then loaded onto a column and washed by gravity flow with 10 column volumes of high salt buffer (25 *mM* Tris-HCl pH 8.0, 500 *mM* NaCl, and 1% sodium cholate) and 10 *mM* imidazole buffer (25 *mM* Tris-HCl pH 8.0, 125 *mM* NaCl, 1% sodium cholate, and 10 *mM* imidazole). Protein was eluted with *Gβγ* lysis buffer supplemented with 1% sodium cholate and 200 *mM* imidazole and concentrated to ~2 *mL* using a 15-mL Amicon concentrator with 30-kD molecular weight cutoff. The final protein was diluted to ~20 *mL* using *Gβγ* lysis buffer supplemented with 1% sodium cholate and the His-YFP was removed by cleavage with 3C PreScission protease overnight. The free His-YFP was removed using TALON resin equilibrated with *Gβγ* lysis buffer supplemented with 1% sodium cholate and 20 *mM* imidazole and the cleaved *Gβγ* was concentrated to 1 *mL* using a 15-*mL* Amicon concentrator with 30-kD molecular weight cutoff. Protein was purified further with size exclusion chromatography using a Superdex 200 10/300 increase column in *Gβγ* lysis buffer supplemented with 1% sodium cholate and 5 *mM* DTT. Fractions containing *Gβγ* were pooled and concentrated to 5-10 mg/mL using 4-*mL* Amicon concentrator with 30-kD molecular weight cutoff and immediately used for reconstitution.

Nonlipidated *Gβγ* was generated by introducing the C68S mutation into the *Gγ* construct, which prevents lipidation ((2, 6). In this work, the YFP was retained on the final protein. Untagged human *Gβ1* was co-expressed with human *Gγ2* C68S with an N-terminal His-YFP tag in High Five cells by infection with 12 and 8 *mL* of P3 baculovirus, respectively. Cells were harvested 36-48 hours after infection by centrifugation at 3,500 x *g* for 15 minutes. Pellets were resuspended in *Gβγ* lysis buffer and lysed by brief sonication. Lysate was clarified by centrifugation for 45 minutes at 39,000 x *g* and bound in batch to TALON resin equilibrated with *Gβγ* lysis buffer. Resin was washed in batch with 10 column volumes of *Gβγ* lysis buffer, loaded onto a column and washed by gravity flow with 10 column volumes of high salt buffer (25 *mM* Tris-HCl pH 8.0 and 500 *mM* NaCl) and 10 *mM* imidazole buffer (25 *mM* Tris-HCl pH 8.0, 125 *mM* NaCl, and 10 *mM* imidazole). Protein was eluted with *Gβγ* lysis buffer supplemented with 200 *mM* imidazole and concentrated to ~10 *mg/mL* using a 15-*mL* Amicon concentrator with 30-kD molecular weight cutoff. *Gβγ*-YFP was purified further via size exclusion chromatography using a Superdex 200 10/300 increase column equilibrated with *Gβγ* lysis buffer supplemented with 5 *mM* DTT. Fractions containing *Gβγ*-YFP were pooled, flash frozen, and stored at -80°C for later use.

For ALFA-nanobody tagged *Gβγ*, the ALFA nanobody was inserted between the N-terminal His-YFP and the human *Gγ2* gene in the background of the C68S mutant. Untagged human *Gβ1* was co-expressed with the ALFA nanobody *Gγ2* construct in High Five insect cells by infection with 12 *mL* P3 baculovirus for each construct. Cells were harvested 36-48 hours after infection by centrifugation at 3,500 x *g* for 15 minutes. Cells were resuspended in *Gβγ* lysis buffer supplemented with 5 *mM* DTT and lysed by brief sonication. Lysate was clarified by centrifugation at 39,000 x *g* for 45 minutes and bound to GFP nanobody-coupled Sepharose resin

equilibrated with $G\beta\gamma$ lysis buffer for one hour. The resin was washed in batch with 10 column volumes of $G\beta\gamma$ lysis buffer then loaded into a column and washed with an additional 10 column volumes by gravity flow. Protein was eluted by cleavage with 3C PreScission protease for two hours, concentrated to 1 *mL* using a 15-*mL* Amicon concentrator with 30-kD molecular weight cutoff, and further purified by size exclusion chromatography using a Superdex 200 10/300 increase column equilibrated with $G\beta\gamma$ lysis buffer supplemented with 5 *mM* DTT. Fractions with nanobody-tagged $G\beta\gamma$ were pooled, flash frozen, and stored at -80°C for later use.

Full-length mouse GIRK2 with a C-terminal ALFA peptide tag upstream of a GFP tag was expressed using HEK293S GnTI$^-$ by infection with 10% (v/v) P3 virus. 10 *mM* sodium butyrate was added 12 hours after infection and the temperature was reduced to 30°C for 48 hours. Cells were harvested by centrifugation at 3,500 x *g* for 15 minutes. Cells were resuspended in GIRK lysis buffer (25 *mM* Tris-HCl pH 7.5, 150 *mM* KCl, and 2 *mM* DTT) and lysed by manual homogenization. Membranes were collected by centrifugation at 39,000 x *g* for 30 minutes, resuspended in fresh GIRK lysis buffer supplemented with protease inhibitors and dNase and manually homogenized. GIRK was extracted from membranes with 1.5% DDM/0.3% CHS for 1.5 hours and the extraction was centrifugated at 39,000 x *g* for 30 minutes. The supernatant was bound to GFP nanobody-coupled Sepharose resin equilibrated with GIRK wash buffer (20 *mM* Tris-HCl pH 7.5, 150 *mM* KCl, 2 *mM* DTT, and 0.05%/0.01% DDM/CHS) for one hour, washed in batch with 10 column volumes of GIRK wash buffer, then loaded into a column and washed with an additional 10 column volumes of GIRK wash buffer by gravity flow. Protein was eluted by cleavage with 3C PreScission protease for 1.5 hours, concentrated to ~10 *mg/mL* using a 15-*mL* Amicon concentrator with 100-kD molecular weight cutoff, and further purified by size exclusion chromatography using a Superose 6 10/300 increase column equilibrated with GIRK SEC buffer (20 *mM* Tris-HCl pH 7.5, 150 *mM* KCl, 10 *mM* DTT, and 0.025%/0.005% DDM/CHS). Fractions with GIRK were pooled, concentrated to ~2 *mg/mL* and used for reconstitution immediately.

Human $G\alpha_q$ was truncated to the methionine at position 7, which prevents palmitoylation (7), and inserted into the pFastBac vector downstream of an 8-His tag and a 3C protease cleavage site. This construct was previously shown to activate $PLC\beta$ enzymes (7-10). Baculovirus was generated according to the manufactures protocol (Invitrogen). $G\alpha_q$ was co-expressed at a 2:1 ratio with untagged rat Ric8A to improve expression (11). High Five insect cells were infected with 7 *mL* of Ric8A and 15 *mL* $G\alpha_q$ p3 virus per liter of culture and harvested after 36-48 hours by centrifugation at 3,500 x *g* for 15 minutes. Cells were resuspended in $G\alpha_q$ lysis buffer (25 *mM* Tris-HCl pH 8.0, 125 *mM* NaCl, 5 *mM* $MgCl_2$, 5 *mM* 2-mercaptoethanol and 10 *mM* imidazole) supplemented with 30 μM GDP and lysed by brief sonication. Lysate was clarified by centrifugation at 39,000 x *g* for 45 minutes and bound in batch to Ni-NTA resin equilibrated with $G\alpha_q$ lysis buffer supplemented with 30 μM GDP. The resin was washed in batch with 10 column volumes of $G\alpha_q$ lysis buffer supplemented with 30 μM GDP, loaded onto a column and washed by gravity flow with 10 column volumes of high salt buffer (25 *mM* Tris-HCl pH 8.0, 500 *mM* NaCl, 5 *mM* $MgCl_2$, 5 *mM* 2-mercaptoethanol, 10 *mM* imidazole and 30 μM GDP), 25 *mM* imidazole buffer (25 *mM* Tris-HCl pH 8.0, 125 *mM* NaCl, 5 *mM* $MgCl_2$, 5 *mM* 2-mercaptoethanol, 25 *mM* imidazole, and 30 μM GDP) and 40 *mM* imidazole buffer (25 *mM* Tris-HCl pH 8.0, 125 *mM* NaCl, 5 *mM* $MgCl_2$, 5 *mM* 2-mercaptoethanol, 40 *mM* imidazole, and 30 μM GDP). Protein was eluted with elution buffer (25 *mM* Tris-HCl pH 8.0, 125 *mM* NaCl, 5 *mM* $MgCl_2$, 5 *mM* 2-mercaptoethanol, 250 *mM* imidazole, and 30 μM GDP) and

concentrated to ~2 *mL* using a 15-*mL* Amicon concentrator with 30-kD molecular weight cutoff and diluted to ~20 *mL* using $G\alpha_q$ dilution buffer (25 *mM* Tris-HCl pH 8.0, 125 *mM* NaCl, 5 *mM* $MgCl_2$, and 5 *mM* 2-mercaptoethanol) supplemented with 30 *μM* GDP. The His tag was cleaved by incubation with 3C PreScission protease for 1.5 hours and separated from the protein using Ni-NTA resin equilibrated with 25 *mM* imidazole buffer. Cleaved $G\alpha_q$ was concentrated to 1 *mL* using a 15-*mL* Amicon concentrator with 30-kD molecular weight cutoff, and further purified by size exclusion chromatography using a Superdex 200 10/300 increase column equilibrated with $G\alpha_q$ SEC buffer (25 *mM* Tris-HCl pH 8.0, 125 *mM* NaCl, 5 *mM* $MgCl_2$, and 5 *mM* DTT) supplemented with 30 *μM* GDP. Fractions with $G\alpha_q$ were pooled, flash frozen, and stored at -80°C for later use.

The Q209L mutation was generated in the same construct using the KLD enzyme mix (NEB). Expression and purification of $G\alpha_q$ Q209L were the same as for the wildtype protein with the following modifications. High Five insect cells were infected with 4 *mL* of Ric8A and 12 *mL* $G\alpha_q$ Q209L P3 virus (3:1 $G\alpha_q$:Ric8A) per liter of culture and harvested after ~36 hours by centrifugation at 3,500 x *g* for 15 minutes. The GDP was replaced with 100 *μM* GTP in all buffers. The GTP-bound state of the protein was confirmed after every purification using the trypsin cleavage assay described below.

For *PLCβ3*·$G\alpha_q$ Q209L complex formation (Fig. S1B), $G\alpha_q$ Q209L with GTP was mixed with *PLCβ3* in a 2:1 molar ratio in buffer containing 25 *mM* Tris-HCl pH 7.4, 125 *mM* NaCl, 3 *mM* $MgCl_2$, 75 *μM* GDP, and 5 *mM* DTT and incubated on ice for one hour. The complex was purified via size exclusion chromatography using a Superdex 200 10/300 Increase column equilibrated with the same buffer.

Trypsin cleavage assay

The nucleotide state (GTP/GDP-AlF_4 or GDP) of $G\alpha_q$ was evaluated using a trypsin cleavage assay as previously described (1). Purified $G\alpha_q$ was mixed with 3 *μg* of trypsin on ice. Samples for SDS-PAGE analysis were removed after 2 minutes and 10 minutes and immediately mixed with SDS loading dye to stop the reaction. Cleavage patterns were evaluated by SDS-PAGE. The GDP-bound state of $G\alpha_q$ produces a band at ~20 kD and the GTP/GDP-AlF_4-bound state produces a band at ~36 kD (1)(Fig. S1C).

Nucleotide exchange of wildtype $G\alpha_q$

For the structure of the *PLCβ3·Gβγ(2)*·$G\alpha_q$ complex and membrane partitioning studies, an additional purification step was added to ensure that all the $G\alpha_q$ was GDP-AlF_4-associated. *Gβγ*-YFP was bound to GFP nanobody-coupled Sepharose resin at 4°C for 2 hours to generate a *Gβγ* column and unbound *Gβγ*-YFP was removed by washing with 10 column volumes of $G\alpha_q$ SEC buffer supplemented with 30 *μM* GDP. Purified $G\alpha_q$ GDP was bound to the column by incubating at 4°C for 2 hours and unbound $G\alpha_q$ was removed by washing with 10 column volumes of $G\alpha_q$ SEC buffer supplemented with 30 *μM* GDP. $G\alpha_q$ GDP-AlF_4 was eluted by incubating the column with GDP-AlF_4 buffer (25 *mM* Tris-HCl pH 8.0, 125 *mM* NaCl, 50 *mM* $MgCl_2$, 10 *mM* NaF, 30 *μM* $AlCl_3$, 30 *μM* GDP, and 5 *mM* DTT) at 30°C for 2-3 hours (12). The GDP-AlF_4 state of $G\alpha_q$ was confirmed using the trypsin cleavage assay.

Protein Reconstitution

For all reconstitutions, lipids in chloroform were mixed and dried under a stream of argon, washed with pentane, dried under a stream of argon and incubated under vacuum overnight. GIRK and lipidated *Gβγ* were reconstituted for bilayer experiments using 3:1 ratio of 1-palmitoyl-2-oleoyl-sn-glycero-3-phosphoethanolamine (POPE): 1-palmitoyl-2-oleoyl-sn-glycero-3-phospho-(1'-rac-glycerol) (POPG). For GIRK reconstitution, lipids were resuspended in 10 *mM* K_2HPO_4, 450 *mM* KCl, and 10 *mM* DTT at 20 *mg/mL* and sonicated to clarity. 1% DM was added to the lipids and the mixture was sonicated again. GIRK was added to the mixture at a protein to lipid ratio of 1:10 (wt/wt), the lipid concentration was diluted to 10 *mg/mL* and incubated at 4°C for one hour. Detergent was removed at 4°C via dialysis against 10 *mM* K_2HPO_4 pH 7.4, 450 *mM* KCl, 10 *mM* DTT, and biobeads. *Gβγ* was reconstituted in the same way but using different buffer conditions for the lipids and dialysis (10 *mM* HEPES pH 7.4, 150 *mM* KCl, and 10 *mM* DTT), 2% sodium cholate instead of DM, and a protein to lipid ratio of 1:5 (wt:wt). In both cases, dialysis buffer was changed every 12 hours for three changes with fresh DTT added at each change. After dialysis, liposomes were incubated with ~50% volume of biobeads for five hours at 4°C, flash frozen, and stored at -80°C until use.

For structural studies and partitioning experiments, liposomes were comprised of a 2:1:1 mixture of 1,2-dioleoyl-sn-glycero-3-phosphoethanolamine (DOPE): 1-palmitoyl-2-oleoyl-glycero-3-phosphocholine (POPC): 1-palmitoyl-2-oleoyl-sn-glycero-3-phospho-L-serine (POPS). In reconstitutions for partition experiments, 0.1 *mol%* 1,2-dioleoyl-sn-glycero-3-phosphoethanolamine-N-(lissamine rhodamine B sulfonyl) (18:1 Liss Rhod PE) was included in the lipid mixture. Lipids were resuspended at 25 *mM* in reconstitution buffer (25 *mM* HEPES pH 7.4, 150 *mM* KCl, 1 *mM* $MgCl_2$, and 5 *mM* DTT) and sonicated to clarity. 40 *mM* sodium cholate was added to the mixture and sonicated briefly. For protein-free liposomes for structural studies, the mixture was diluted to 20 *mM* lipid maintaining the 40 *mM* sodium cholate and incubated at 4°C for one hour. For *Gβγ*-containing liposomes, *Gβγ* was added at a protein to lipid ratio of 1:15 (wt/wt) and the lipid concentration was reduced to 20 *mM* maintaining 40 *mM* sodium cholate and incubated at 4°C for one hour. For protein-free liposomes for partitioning experiments, lipids were diluted to 10 *mM,* maintaining the 40 *mM* sodium cholate and incubated at 4°C for one hour. For all samples, detergent was removed using four exchanges of 200 *mg/mL* biobeads washed with reconstitution buffer after two hours, 12 hours, two hours, and two hours at 4°C. Liposomes for structural studies were used immediately following reconstitution to prepare grids. Liposomes for partitioning studies were flash frozen and stored at -80°C until use.

Bilayer experiments and analysis

Experiments were carried out and analyzed as previously described (2). 2DOPE:1POPC:1POPS (wt:wt:wt) lipids supplemented with 1 *mol%* 1,2-dioleoyl-sn-glycero-3-phospho-(1'-myo-inositol-4',5'-bisphosphate) (*PIP2*) were used for all experiments. Lipids in chloroform were dried under a stream of argon and resuspended at 22 *mg/mL* in decane. Two 3-*mL* chamber cups in a vertical bilayer configuration were connected by a 100 *μm* thick piece of Fluorinated ethylene propylene copolymer with a ~250 *μm* hole, which was used to paint a bilayer with the lipid-decane mixture. Bilayer buffer (25 *mM* HEPES pH 7.4, 150 *mM* KCl, 30 *mM* NaCl, 2 *mM* $MgCl_2$, and 100 *μM* $CaCl_2$) supplemented with 100 *μM* GTP was used in both chambers for all experiments.

The Reference electrode and the Ground electrode were connected to the Trans and Cis chambers respectively via agarose salt bridges. A magnetic stir bar was included in the Cis chamber to facilitate continuous mixing; therefore all components of the experiment were added to the Cis chamber. Voltage across the lipid bilayer was controlled with an Axopatch 200Bamplifier in whole-cell mode. The analog current signal was lowpass filtered at 1 kHz (Bessel) and digitized at 10 kHz with a Digidata 1440A digitizer. Digitized data were recorded with the software pClamp (MolecularDevices). At +80 mV, current from channels with their *PIP2*-binding sites in the Trans chamber, which is inaccessible to *PLCβ3* added to the Cis chamber, was blocked by Mg^{2+} (2). Therefore, current measured at +80 mV is only from channels sensitive to *PLCβ3*-dependent degradation of *PIP2*, allowing the current to decay completely.

$G\alpha_q$ Q209L with GTP was used in all bilayer experiments. 30 *nM* ALFA nanobody-tagged *Gβγ* was added to the Cis chamber at the beginning of each experiment. GIRK or *Gβγ*-containing liposomes were supplemented with KCl to a final concentration of 1 *M*, sonicated briefly at room temperature, and fused to the membrane from the Cis chamber. After fusing vesicles with GIRK, baseline current at +80 mV was measured for 3-5 minutes. Then the desired concentration of $G\alpha_q$ was added to the Cis chamber under continuous mixing and did not alter the baseline GIRK current (Fig. S1D). After at least 1 minute, 29 *nM PLCβ3* for wildtype enzyme was added to the Cis chamber under continuous mixing while recording. For experiments with wildtype *PLCβ3* and *Gβγ* and $G\alpha_q$, *Gβγ* was incorporated into the bilayer via vesicle fusion and the $PLC\beta3{\cdot}G\alpha_q$ complex was pre-formed on ice and added to the Cis chamber under continuous mixing to initiate the current decay. For experiments with *PLCβ3* ΔX-Y contact or ΔX-Y all constructs, a final concentration of 290 *pM PLCβ3* was used. In the presence of $G\alpha_q$, the $PLC\beta3{\cdot}G\alpha_q$ complex was pre-formed on ice and added to the Cis chamber under continuous mixing to initiate the current decay. After all current decays, a voltage family was measured to ensure integrity of the bilayer and then saturating C8PIP2 (32 *μM*) was added to recover the GIRK current. Any experiment where the current did not recover was discarded. We are confident that the starting *PIP2* concentration in the membrane was not affected by vesicle fusion because the kinetics of PIP2 hydrolysis are not dependent on the number of channels that fused (2).

Analysis was carried out in Clampfit and qtiplot. To reduce the amplitude of high-frequency undesired signal owing largely to the stir bar in the recording chamber, current time series were low pass filtered at 10-20 Hz and exported to qtiplot. Decays lasting more than 100 s, i.e., at low concentrations of $G\alpha_q$, were down sampled by a factor of 10 prior to exporting. We also analyzed our fastest decays without low pass filtering to ensure that the 10-20 Hz filter did not alter the determination of V_{max} and K_M to a significant degree (i.e., 10-20 Hz did not over filter the kinetic process under study). In qtipliot, decays were leak subtracted (where the baseline current remaining in the bilayer at the end of the decay, I_{leak}, was subtracted from each point in the decay) and normalized to the starting GIRK current scaled according to the starting PIP2 concentration, 1.0 *mol%*, which is ~30% of the GIRK current at saturating *PIP2* concentrations (Fig. S2A).

To test the effects of *DAG* (Fig. S3), 1,2-Dioleoyl-rac-glycerol was mixed with bilayer lipids at 1.0 *mol%* and used to paint bilayers. This is the maximum concentration that would be present in our experiments after all the *PIP2* is hydrolyzed. To test the effects of *IP3* (Fig. S3), D-myo-Inositol 1,4,5-tris-phosphate trisodium salt was solubilized in water and added to the bilayer chamber at a final concentration of 1.0 *μM*. This concentration is several orders of

magnitude higher than the maximal concentration of *IP3* estimated to be present in the chamber after all the *PIP2* has been hydrolyzed (~50 *pM*). The higher concentration should account for any possible higher local concentration present at the membrane following catalysis.

K_M and V_{max} for each experiment were determined as previously described (2). Briefly, we determined the relationship between normalized GIRK current, I/I_{max}, and *PIP2* concentration in the bilayer using *PIP2* titration experiments. The relationship is described by SI Appendix, Eq. S1,

$$\frac{I}{I_{max}} = A\frac{[PIP2]^r}{k^r+[PIP2]^r} \quad \text{(S1)}$$

where A=0.88, k=1.5, and r=2.2 (2). We observed that *PLCβ3* operates according to Michaelis-Menten kinetics (Fig. 1D), described by Eq. 1 in the main text. The *PIP2* concentration as function of time was determined by integrating Eq. 1 from τ = 0 to τ = t, (Eq. 2 in the main text).

$$[PIP2(t)] = K_M ProductLog\ \frac{e^{\frac{([PIP2(0)]-tV_{max})}{K_M}}[PIP2(0)]}{K_M}. \quad \text{(S2)}$$

Eq. 2 was substituted into SI Appendix, Eq. S1 and an additional term, C, was added to capture imperfections in the leak subtraction, yielding SI Appendix, Eq. S3,

$$\frac{I}{Imax} = C + A\frac{\left(K_M\, ProductLog\,\frac{e^{\frac{([PIP2(0)]-tV_{max})}{K_M}}[PIP2(0)]}{K_M}\right)^r}{k^r+\left(K_M\, ProductLog\,\frac{e^{\frac{([PIP2(0)]-tV_{max})}{K_M}}[PIP2(0)]}{K_M}\right)^r}. \quad \text{(S3)}$$

Normalized current decays were fit to SI Appendix, Eq. S3 to determine V_{max}, K_M, and C, which was very small, < 1% of the normalized current.

NMR experiments to measure lipid concentration

10-20 *µL* from each reconstitution was dissolved in ~550 *uL* of a mixture of deuterated methanol and chloroform (5:1) containing 100 *µM* of the standard sodium trimethylsilyl propionate (TSP) in 5 *mm* tubes. Proton spectra were measured on a Bruker 600 MHz instrument with a 5 *mm* HCN cryoprobe and an AVANCE NEO console. Spectra were collected at 298 K using a 30° flip angle, 16 scans, and 2.8 second acquisition time and a recycle delay of 18 µs. Spectra were processed using TopSpin 4.1.1 for phasing, baseline correction and line broadening. The lipid -CH_3 peak at 0.875 ppm was integrated relative to the TSP peak at 0 ppm and normalized to the difference in protons (9 for TSP and 6 for the lipid CH_3) (2). The normalized peak area was used to determine the lipid concentration using the known concentration of TSP.

PLCβ3 and $G\alpha_q$ vesicle partition experiments

Reconstituted liposomes were subjected to 10 freeze-and-thaw cycles and extruded 21 times through a 200 *nm* membrane to produce LUVs. The reported lipid concentration is 50% of the total lipid concentration added in solution because partitioning proteins can only access the outer

leaflet. Fixed concentrations of lipids were mixed with proteins of interest (wildtype $G\alpha_q$ alone, LD655 labeled *PLCβ3* ΔX-Y all alone, or wildtype LD655 labeled-*PLCβ3* and 200 *nM* wildtype $G\alpha_q$ or 200 *nM* $G\alpha_q$ Q209L) in partitioning buffer (25 *mM* HEPES pH 7.4, 150 *mM* KCl, 1 *mM* $MgCl_2$, and 5 *mM* DTT) supplemented with 10 *mM* NaF, 30 *μM* GDP, and 30 *μM* $AlCl_3$ for wildtype $G\alpha_q$ or 100 *μM* GTP for $G\alpha_q$ Q209L. Lipid-protein mixtures were incubated for one hour and centrifuged for one hour at 100,000 x *g* at room temperature. The supernatant was removed, and the membrane pellet was resuspended in an equal volume of buffer. The input, pellet, and supernatant samples were analyzed by SDS-PAGE. Samples with $G\alpha_q$ alone were stained using the Thermo Scientific silver stain kit and samples with *PLCβ3* were imaged using in-gel fluorescence to detect LD655-labeled *PLCβ3* (Fig. S4A-D). We previously showed that LD655-labeled *PLCβ3* has the same partition coefficient and unlabeled *PLCβ3* (2). Gel bands were quantified using Bio-Rad imagelab software. For experiments with *PLCβ3*, input, supernatant, and pellet samples were solubilized in 5% Anapoe-C12E10 to eliminate scattering artifacts and the LD655 fluorescence (ext-649, em-666) and Rhodamine fluorescence (ext-560, em-583) were measured using a Tecan plate reader. The Rhodamine signal was used to estimate the fraction of lipids that were pelleted, and the measurements were corrected for this as well as the loss of material using the difference between the input and output (pellet and supernatant) LD655 signal. Each lipid concentration was repeated with two different protein concentrations, 400 and 200 *nM* for $G\alpha_q$ alone, 400 *nM* and 300 *nM* for *PLCβ3* ΔX-Y constructs, and 200 *nM* and 100 *nM* for *PLCβ3*. Values for fraction of protein partitioned (F_p), were determined, plotted against lipid concentration, and fit to Eq. 5 to determine K_x (Fig. 2D, S4F-G). For experiments with *PLCβ3*, values from the gels and the solution fluorescence measurements were consistent. The values from the solution measurements are reported.

Cryo-EM sample preparation and data collection

For the $PLC\beta3{\cdot}G\alpha_q$ complex with reconstituted liposomes, $G\alpha_q$ was incubated on ice for 30 minutes in the presence of GDP-AlF_4 (25 *mM* HEPES pH 7.4, 150 *mM* KCl, 5 *mM* $MgCl_2$, 0.9 *mM* $CaCl_2$, 10 *mM* NaF, 50 *μM* GDP, 30 *μM* $AlCl_3$, and 5 *mM* DTT), then mixed with *PLCβ3* in a 2:1 molar ratio and incubated on ice for one hour. The complex was purified via size exclusion chromatography using a Superdex 200 10/300 Increase column equilibrated with 25 *mM* HEPES pH 7.4, 150 *mM* KCl, 5 *mM* $MgCl_2$, 0.9 *mM* $CaCl_2$, 10 *mM* NaF, 50 *μM* GDP, 30 *μM* $AlCl_3$, and 5 *mM* DTT (Fig. S1A). Purified $PLC\beta3{\cdot}G\alpha_q$ complex was mixed with vesicles at final concentrations of 3.6 *μM* complex and 17.5 *mM* lipids and incubated at room temperature for one hour. For the $PLC\beta3{\cdot}G\beta\gamma(2){\cdot}G\alpha_q$ complex, $G\alpha_q$ GDP-AlF_4 was mixed with *PLCβ3* in a 2:1 molar ratio, incubated on ice for one hour and exchanged into buffer containing 25 *mM* HEPES pH 7.4, 150 *mM* KCl, 5 *mM* $MgCl_2$, 0.9 *mM* $CaCl_2$, 10 *mM* NaF, 50 *μM* GDP, 30 *μM* $AlCl_3$, and 5 *mM* DTT using a PD10 desalting column. Purified $PLC\beta3{\cdot}G\alpha_q$ complex was mixed with *Gβγ*-containing vesicles (reconstituted at 1:15 wt/wt) at final concentrations of 4.1 *μM* complex and 16.7 *mM* lipids and incubated at room temperature for one hour.

Both samples were supplemented with 3 *mM* Fluorinated Fos-Choline-8 ~5 minutes before grid preparation. Quantifoil R1.2/1.3 400 mesh holey carbon Au grids were glow discharged for 20s, and 3.5 *μL* of sample was applied, incubated for 5 minutes at 22°C and 100% humidity, and manually blotted from below. An additional 3.5 *μL* of sample was applied and after 30 seconds of incubation, grids were blotted for 3.5s with a blot force of 0 and plunge

frozen in liquid ethane using a FEI Vitrobot Mark IV. For data acquisition, grids were loaded onto a 300-kV Titan Krios transmission electron microscope, located at the HHMI Janelia Research Campus, with a Gatan K3 Summit direct electron detector and a GIF quantum energy filter with a slit width of 20 eV. 25,668 movies were collected for the $PLC\beta3{\cdot}G\alpha_q$ complex and 29,832 movies were collected for the $PLC\beta3{\cdot}G\beta\gamma(2){\cdot}G\alpha_q$ complex in superresolution mode with a pixel size of 0.4195 Å and a defocus range of 1.5 to 2.5 μm using SerialEM (13). The movies were recorded with 50 frames, a total dose of 60 $e^-/Å^2$ (1.2 $e^-/Å^2$/frame) and a 4.26 second total exposure time (0.085s/frame) for the $PLC\beta3{\cdot}G\alpha_q$ complex or a 3.86 second total exposure time (0.071s/frame) for the $PLC\beta3{\cdot}G\beta\gamma(2){\cdot}G\alpha_q$ complex.

Cryo-EM data processing

For both complexes, motion correction was performed with 2x binning using the RELION implementation (in RELION 3.1) and CTF estimation was carried out using CTFfind4 (14-16). Particle picking was caried out using the model trained for PLCβ3 on vesicles in crYOLO (2, 17). For the $PLC\beta3{\cdot}G\alpha_q$ complex, 3,543,739 particles were picked and extracted with 2x binning and a 260 Å box size, sorted to 2,825,696 using a resolution cutoff of 4 Å, and sorted to 2,342,176 using 2D classification in cryoSPARC. Iterative rounds of *ab initio* reconstruction and heterogenous refinement in cryoSPARC were carried out to generate an initial reconstruction with density for the membrane and protein protruding (Fig. S5F). This map was used as an input for heterogenous refinement in cryoSPARC to sort particles based on membrane alignment. 592,280 particles with good membrane alignment were selected, subjected to refinement in RELION, and signal subtraction was applied to remove the membrane density. Subtracted particles were subjected to 2D classification in cryoSPARC and particles from the best 2D classes, 213,197, were used for *ab initio* reconstruction to obtain an initial map resembling the $PLC\beta3{\cdot}G\alpha_q$ complex. All subtracted particles were subjected to iterative heterogenous refinement using this map as input. A final subset of 229,523 particles yielding a reconstruction with clear secondary structure features was un-subtracted and re-extracted without binning and a 289 Å box size. These particles were subjected to several cycles of Bayesian polishing and CTF refinement in RELION (18, 19) and local refinement with a mask on the entire complex in cryoSPARC. After polishing, 3D classification without alignment was carried out to improve the resolution, search for alternate configurations of the X-Y linker and obtain density for the membrane. The best subset contained 67,454 particles and resulted in a 3.4 Å reconstruction from local refinement in cryoSPARC. Two subsets containing 20,261 and 15,486 particles yielded reconstructions with density for the membrane (Fig. 6B-C). No reconstructions with differing density for the X-Y linker were observed.

For the $PLC\beta3{\cdot}G\beta\gamma(2){\cdot}G\alpha_q$ complex, 5,332,307 particles were picked and extracted with 2x binning and a 289 Å box size, sorted to 5,128,184 particles using a resolution cutoff of 5 Å, and sorted to 4,917,142 particles using 2D classification in cryoSPARC. 271,175 particles from the best 2D classes were subjected to *ab initio* reconstruction and a map with density for the membrane and two blobs of protein protruding was produced. This reconstruction was used as an input for heterogenous refinement on all 4,917,142 particles to sort based on membrane alignment. Three classes with 1,120,862, 1,235,752, and 1,682,270 particles (4,038,884 particles total) were refined separately in RELION and subjected to signal subtraction to remove the membrane density. Subtracted particles were subjected to 2D classification in cryoSPARC. 195,750 particles from the best 2D classes were subjected to *ab initio* reconstruction to obtain a

map resembling the *PLCβ3·Gβγ(2)·Gα*$_q$ complex. This map was used as an input for iterative heterogenous refinement on all 4,038,884 subtracted particles. 484,174 particles yielding a reconstruction with clear secondary structure features were un-subtracted and re-extracted without binning and a 289 Å box size. These particles were subjected to several rounds of Bayesian polishing and CTF refinement in RELION (18, 19) and local refinement with a mask on the entire complex in cryoSPARC. After polishing, 3D classification without alignment was carried out to improve the resolution, search for alternate configurations of the X-Y linker and obtain density for the membrane. The best subset contained 359,215 particles and resulted in a 3.4 Å reconstruction from local refinement in cryoSPARC. Six subsets containing 19,266, 15,138, 27,763, 21,151, 17355, and 12,194 particles yielded reconstructions with density for the membrane (Fig. 6D). No reconstructions with differing density for the X-Y linker were observed. A subset of 48,367 particles was identified that only contained density for *PLCβ3* and *Gβγ*. Refinement of these particles yielded a reconstruction that was very similar to the previously reported *PLCβ3-Gβγ* complex and these particles were excluded from the final subset.

Model building and Validation

For the *PLCβ3·Gα*$_q$ complex, a previously determined crystal structure was used as the starting model (PDBID 4GNK, (10)). It was fit into the density and refined with PHENIX real-space refine (20) and manually inspected and adjusted where necessary. Regions with poor or weak density were removed. The final model consists of *PLCβ3*: 13-33, 38-92, 97-470, and 575-878 with the side chains of E14, R23, R24, M59, E60, K85, E88, E100, K169, K173, E187, R185, F197, K228, R268, K761, D777, D850, Q853, Y855, R872, R874, and Q875 truncated due poor density, and *Gα*$_q$: 38-352 with the side chains of K98, Y103, K120, N126, D165, R166, Q197, Q265, and K276 truncated due to poor density. For the *PLCβ3·Gβγ(2)·Gα*$_q$ complex, the *Gβγs* from the *PLCβ3-Gβγ* complex on vesicles (PDBID: 8EMW, (2) were merged with the *PLCβ3·Gα*$_q$ complex as a starting model. It was fit into the density and refined with PHENIX real-space refine (20) and manually inspected and adjusted where necessary. Regions with poor or weak density were removed. The final model consists of *PLCβ3*: 13-92, 99-470, 575-873, with the sidechains of R82, K85, E88, E100, K129, K173, E191, E207, E211, K236, K238, K268, E303, M383, D417, K420, K601, D721, E779, D850, Q853, Y855, and R872 removed due to poor density, *Gα*$_q$: 38-352 with the sidechains of E49, D69, E70, R73, K98, E104, E115, D117, E125, D155, D165, D169, Q197, Q265, D296, D301, E307, and D321 truncated due to poor density. *Gβ*1: 6-126 and 133-340, with sidechains from L7, R8, Q9, K15, R19, D20, R22, K23, N36, R46, R52, D66, K89, R96, R134, R137, R214, E215, E226, K280, and R283 truncated due to poor density, *Gγ*1: 9-61, with the side chains of K20, M21, K29, E47, and E58 truncated due to poor density, *Gβ*2: 4-126, and 133-340 with sidechains from L7, Q9, R19, K23, E27, Q32, N35, N36, R42, R46, D66, R68, N88, R96, R134, R137, D154, E172, Q175, R214, E215, M216, R251, D258, Q259, K280, K301, D322, and K337 truncated due to poor density, and *Gγ*2: 8-51 with sidechains from Q11, R13, K14, M21, N24, K29, and K46 truncated due to poor density. Model quality was assessed with validation in Phenix using MolProbity score (21) and geometry evaluation (Table S1). Figures were made using ChimeraX (22, 23).

Appendix 1: The integrated Michaelis-Menten rate equation.

We did not invent the analysis of enzyme kinetics using the time evolution of product appearance (or substrate disappearance). Indeed, Michaelis and Menten in their original study of invertase, and many others since, analyzed the time course of enzyme activity, but originally with a different mathematical formalism because modern numerical integration methods were not available (24, 25). The Michaelis-Menten rate equation has been popularized and is the approach almost exclusively taught in books and classrooms. For the approach we have taken, we thought it should be sufficient to reference excellent published explanations (24, 25), however, recurrent misunderstanding during the review process of our work on this subject prompted us to demonstrate here with a simple example of how it works. This exercise is not intended to be a further analysis of our data, but a simple demonstration of how, for the system and conditions we are studying, the integrated Michaelis-Menten equation allows us to approximate K_M and V_{max} (and therefore, with independent knowledge of enzyme concentration, which we have, k_{cat}). We begin with the reaction scheme:

$$E + S \underset{k_b}{\overset{k_f}{\rightleftharpoons}} E \cdot S \xrightarrow{k_{cat}} E + P.$$

In our case E is *PLCβ3*, S is *PIP2*, $E \cdot S$ stands for a molecular complex composed of one E and one S molecule, and P stands for the products *IP3* and *DAG*. The evolution of this system from $t = 0$ is given by the solution of the equations:

$$\frac{dS[t]}{dt} = -k_f S[t]E[t] + k_b E \cdot S[t]$$

$$\frac{d(E \cdot S[t])}{dt} = k_f S[t]E[t] - (k_b + k_{cat})E \cdot S[t]$$

$$E[t] + E \cdot S[t] = E_{tot}\ ,$$

with initial conditions $S[0] = 1$, $E[0] = E_{tot}$. We refer to the solution of these equations for $S[t]$ (i.e., the *PIP2* concentration as a function of time) as the 'true' solution.

Next, consider the approach we take in the main text of this paper. We express the rate of substrate (*PIP2*) disappearance (requiring a minus sign) using the Michaelis-Menten rate equation,

$$v = \frac{dS[t]}{dt} = -\frac{V_{max}\, S[t]}{K_M + S[t]},$$

which, for $K_M = (k_b + k_{cat})/k_f$ and $V_{max} = k_{cat}\ E_{tot}$, can be calculated for the reaction scheme above if $k_f S[t]E[t] = (k_b + k_{cat})E \cdot S[t]$. We then integrate v from time 0 to t to find

$$S[t] = K_M ProductLog\ \frac{e^{\frac{([S[0]]-tV_{max})}{K_M}}\ S[0]}{K_M}.$$

We refer to the above for *S[t]* as the 'approximate' solution. Next, we assign rate constant values, calculate the 'true' solution (i.e., by solving the above rate equations) and then we fit *S[t]* to the 'true' solution to get an 'approximate' solution. For k_f = 10,000 ($mol\%\ s$)$^{-1}$, k_b = 4000 s^{-1}, with k_{cat} = 1.7 s^{-1} and E_{tot} = 0.0015 *mol%* (approximating wild type, panel A), with k_{cat} = 2000 s^{-1} and E_{tot} = 0.000015 *mol%* (approximating an X-Y mutant *PLCβ3,* panel B) or with k_{cat} = 6.6 s^{-1} and E_{tot} = 0.045 (approximating saturating $G\beta\gamma$ + 10 *nM* $G\alpha_q$, *panel C*) the results are as follows (black curves denote 'true' solutions and red dashed curves denote the fitted, approximate solutions):

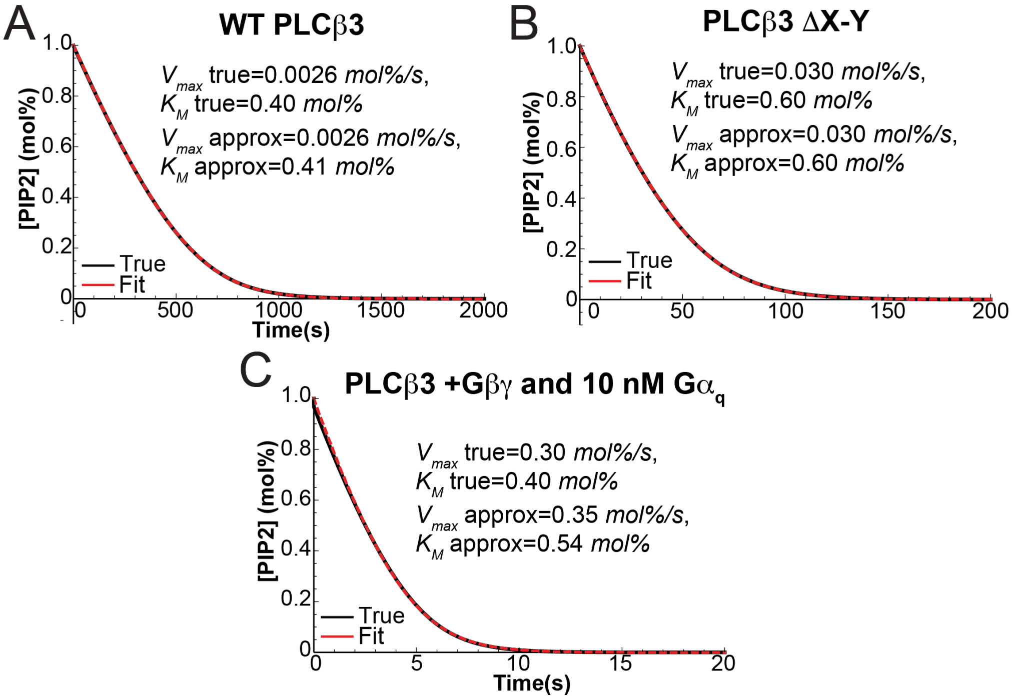

The integrated form of the Michaelis-Menten equation (Lambert W Function) approximates the 'true' process (as described by the reaction equations above that define the system) for a wide range of k_f, k_b and k_{cat} values. As when using the Michaelis-Menten initial rate equation, it is important that $E_{tot} \ll S[0] + K_M$, which is the case for our studies. For the conditions shown in Panel C, there is a deviation between the fitted and true values of K_M and V_{max}. These conditions correspond to our experiments in which a saturating quantity of $G\beta\gamma$ is used, which increases the local enzyme concentration. Even in this most extreme case, $E_{tot} \ll S[0] + K_M$ remains true, and the expected inaccuracy is small, likely within the uncertainty of our experimental data.

The reader should realize that the only real difference between measuring initial rates at various substrate concentrations and measuring the slope along the decay curve (which is then paired with the substrate concentration on the Y-axis) is the presence of product in the latter and the absence of product in the former. These approaches should give the same

result if the products do not alter the function of the enzyme. For *PLCβ3* we do not observe product inhibition (Fig. S3).

Appendix 2: A calculation to explore whether the high k_{cat} in the X-Y linker deletions renders $PLC\beta 3$ insensitive to $G\alpha_q$ regulation.

In this study we demonstrated that $G\alpha_q$ increases k_{cat} by about 35-fold. We further demonstrated that when the X-Y linker is removed by mutation, $G\alpha_q$ no longer causes the 35-fold enhancement. From these observations, we hypothesized that the effect of $G\alpha_q$ to increase k_{cat} is somehow mediated by the X-Y linker. Because X-Y linker removal increases k_{cat} from 1.7 s^{-1} to ~ 2000 s^{-1}, here we ask the question, could the mutant $PLC\beta 3$ have entered a diffusion-limited realm? In this case enzyme turnover would become insensitive to k_{cat} enhancement owing to a shift in the 'rate limiting step' to diffusion of substrate up to the active site, and our hypothesis that $G\alpha_q$ regulates the active site would be incorrect. To assess the likelihood of this possibility, we present the following calculation.

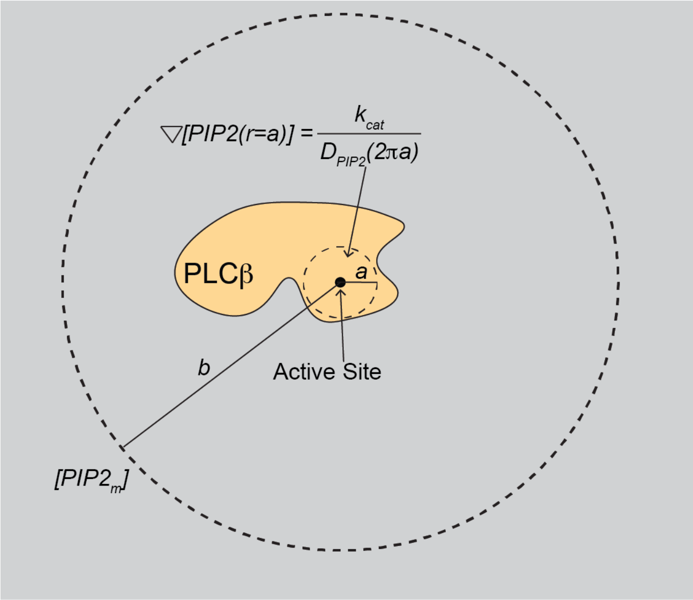

Imagine a $PLC\beta 3$ enzyme on the membrane surface and define an imaginary circle around its active site with a radius $a = 1$ nm (shown above). Given the concentration of $PIP2$ in the membrane and its diffusion coefficient D_{PIP2} (~ 1 μm^2 s^{-1}) we can calculate the concentration profile of $PIP2$ as a function of radial distance r away from the active site when k_{cat} equals its value in the absence of the X-Y linker, say 2000 s^{-1}, to assess whether $PIP2$ becomes depleted near the active site, i.e., diffusion limited. An important assumption must be made in this calculation concerning boundary conditions on steady state diffusion in 2 dimensions. We note that $PLC\beta 3$ also diffuses (say, with $D_{PLC\beta 3} = 1$ μm^2 s^{-1}), and therefore keeps moving a distance approximately 63 nm every 1 ms. Our assumption is, $PLC\beta 3$ 'refreshes' its concentration at boundary b, 63 nm away. Thus, we will create an imagined steady state $PIP2$ concentration profile as a function of distance r away from the active site, between 'bulk' membrane concentration at b and a gradient of concentration at a that will yield a net inward flux of $PLC\beta 3$ equal to k_{cat}. The concentration profile is given by solving

$$\nabla^2[PIP2(r)] = 0$$

subject to the boundary conditions

$$\nabla[PIP2(r = a)] = \frac{k_{cat}}{D_{PIP2}(2\,\pi\,a)}, \qquad [PIP2(r = b)] = [PIP2_m],$$

which gives

$$[PIP2(r)] = \frac{2\,\pi\,D_{PIP2}\,[PIP2_m] + kcat(Log[r] - Log[b])}{2\,\pi\,D_{PIP2}}.$$

Solutions to this expression are shown in the two graphs below.

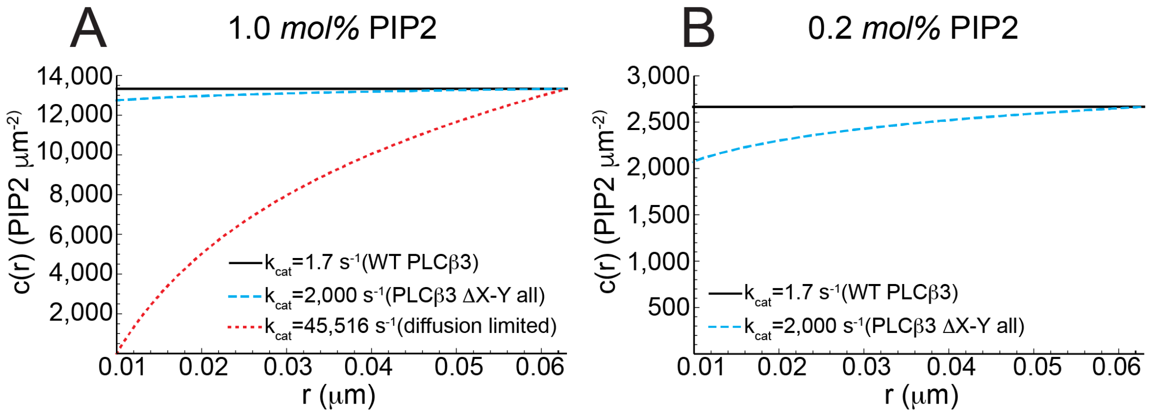

Panel A shows, with 1.0 *mol% [PIP2$_m$]*(~13,333 *PIP2* μm^{-2}), the calculated concentration profile for k_{cat} = 1.7 s^{-1} (black), 2000 s^{-1} (blue) and 45,516 s^{-1} (red, the diffusion limited case, corresponding to the boundary condition $[PIP2(r = a)] = 0$) with D_{PIP2} = 1.0 $\mu m^2\ s^{-1}$. At 2000 s^{-1}, minimal depletion of substrate occurs. Panel B shows, with 0.2 *mol% [PIP2$_m$]*, the concentration profile for k_{cat} = 1.7 s^{-1} (black) and 2000 s^{-1} (blue). Even at this lower *PIP2* concentration, the reaction is far from diffusion limited. While several assumptions are made in this calculation, it presents a rational argument that the *PLCβ3* reaction under our assay conditions are far from diffusion limited. While a rate increase from 2000 s^{-1} to 35 times 2000 s^{-1} (The fold activation by $G\alpha_q$) is not possible based on the diffusion limited example above, we should see a substantial rate increase, but we do not. For this reason, we hypothesize that the increased k_{cat}, from 1.7 s^{-1} to ~60 s^{-1}, due to binding of $G\alpha_q$ to *PLCβ3,* results from allosteric regulation mediated at least in part through the X-Y linker.

SI References

1. E. P. Marin *et al.*, The Function of Interdomain Interactions in Controlling Nucleotide Exchange Rates in Transducin. *Journal of Biological Chemistry* **276**, 23873-23880 (2001).
2. Maria E. Falzone, R. MacKinnon, *Gβγ* activates *PIP2* hydrolysis by recruiting and orienting *PLCβ* on the membrane surface. *Proceedings of the National Academy of Sciences* **120**, e2301121120 (2023).
3. Y. Z. Tan *et al.*, Addressing preferred specimen orientation in single-particle cryo-EM through tilting. *Nature Methods* **14**, 793-796 (2017).
4. T. H. Charpentier *et al.*, Membrane-induced allosteric control of phospholipase C-β isozymes. *Journal of Biological Chemistry* **289**, 29545-29557 (2014).
5. Q. Zheng *et al.*, Electronic tuning of self-healing fluorophores for live-cell and single-molecule imaging. *Chem. Sci.* **8**, 755-762 (2016).
6. C. Zhao, R. MacKinnon, Structural and functional analyses of a GPCR-inhibited ion channel TRPM3. *Neuron* **0** (2022).
7. J. R. Hepler *et al.*, Functional importance of the amino terminus of Gqα. *Journal of Biological Chemistry* **271**, 496-504 (1996).
8. A. M. Lyon *et al.*, An autoinhibitory helix in the C-terminal region of phospholipase C-β mediates Gαq activation. *Nature Structural and Molecular Biology* **18**, 999-1005 (2011).
9. A. M. Lyon, J. A. Begley, T. D. Manett, J. J. G. Tesmer, Molecular mechanisms of phospholipase C β3 autoinhibition. *Structure* **22**, 1844-1854 (2014).
10. A. M. Lyon, S. Dutta, C. A. Boguth, G. Skiniotis, J. J. G. Tesmer, Full-length Gαq-phospholipase C-β3 structure reveals interfaces of the C-terminal coiled-coil domain. *Nature Structural and Molecular Biology* **20**, 355-362 (2013).
11. P. Y. Chan *et al.*, Purification of heterotrimeric G protein α subunits by GST-Ric-8 association: Primary characterization of purified Gαolf. *Journal of Biological Chemistry* **286**, 2625-2635 (2011).
12. P. Chidiac, V. S. Markin, E. M. Ross, Kinetic control of guanine nucleotide binding to soluble Gα(q). *Biochemical Pharmacology* **58**, 39-48 (1999).
13. D. N. Mastronarde, Automated electron microscope tomography using robust prediction of specimen movements. *Journal of Structural Biology* **152**, 36-51 (2005).
14. J. Zivanov *et al.*, New tools for automated high-resolution cryo-EM structure determination in RELION-3. *eLife* **7**, e42166 (2018).
15. A. Rohou, N. Grigorieff, CTFFIND4: Fast and accurate defocus estimation from electron micrographs. *Journal of Structural Biology* **192**, 216-221 (2015).
16. S. Q. Zheng *et al.*, MotionCor2: Anisotropic correction of beam-induced motion for improved cryo-electron microscopy. *Nature Methods* **14**, 331-332 (2017).
17. T. Wagner *et al.*, SPHIRE-crYOLO is a fast and accurate fully automated particle picker for cryo-EM. *Communications Biology* **2**, 1-13 (2019).
18. J. Zivanov, T. Nakane, S. H. W. Scheres, A Bayesian approach to beam-induced motion correction in cryo-EM single-particle analysis. *IUCrJ* **6**, 5-17 (2019).
19. J. Zivanov *et al.*, RELION-3 : new tools for automated high-resolution cryo-EM structure determination. *bioRxiv* 10.1101/421123, 1-38 (2018).
20. P. V. Afonine, J. J. Headd, T. C. Terwilliger, P. D. Adams, PHENIX News. *Computational Crystallography Newsletter* **4**, 43-44 (2013).

21. V. B. Chen *et al.*, MolProbity: All-atom structure validation for macromolecular crystallography. *Acta Crystallographica Section D: Biological Crystallography* **66**, 12-21 (2010).
22. E. F. Pettersen *et al.*, UCSF Chimera - A visualization system for exploratory research and analysis. *Journal of Computational Chemistry* **25**, 1605-1612 (2004).
23. E. F. Pettersen *et al.*, UCSF ChimeraX: Structure visualization for researchers, educators, and developers. *Protein Science* **30**, 70-82 (2021).
24. M. Goličnik, The integrated Michaelis-Menten rate equation: <i>déjà vu</i> or <i>vu jàdé</i> ? *Journal of Enzyme Inhibition and Medicinal Chemistry* **28**, 879-893 (2013).
25. K. A. Johnson, R. S. Goody, The Original Michaelis Constant: Translation of the 1913 Michaelis–Menten Paper. *Biochemistry* **50**, 8264-8269 (2011).
